# Offering mental health first aid to a person experiencing psychosis: a Delphi study to redevelop the guidelines published in 2008

**DOI:** 10.1186/s40359-021-00532-7

**Published:** 2021-02-12

**Authors:** Fairlie A. Cottrill, Kathy S. Bond, Fiona L. Blee, Claire M. Kelly, Betty A. Kitchener, Anthony F. Jorm, Nicola J. Reavley

**Affiliations:** 1 Mental Health First Aid International, Parkville, VIC Australia; 2 Centre for Mental Health, Melbourne School of Population and Global Health, University of Melbourne, Parkville, VIC Australia; 3 Kitchener Consulting, Melbourne, Australia

**Keywords:** Mental health first aid, Psychosis, Delphi study, Mental illness, Mental health, Expert consensus, Community guidelines

## Abstract

**Background:**

Psychotic illnesses can have a major impact on those who experience them. Timely treatment for psychosis is important and friends, family members and the public can be a facilitating factor in social support and professional help-seeking. Expert consensus guidelines on how to provide mental health first aid to a person experiencing psychosis were developed in 2008. This Delphi study aimed to redevelop the guidelines to reflect current evidence.

**Methods:**

The Delphi consensus method was used to determine which helping strategies should be included in the redeveloped guidelines. A systematic search of grey and academic literature was undertaken to identify strategies on how a member of the public can assist someone experiencing psychosis. These strategies were organised into questionnaire statements. Two expert panels—one comprising people with lived experience (Carers and Consumers) and one of professionals—completed three consecutive rounds of online survey questionnaires to rate the importance of each helping statement for inclusion in the guidelines. Statements were included in the guidelines if they were endorsed by at least 80% of each panel.

**Results:**

The expert panels rated 515 statements across three rounds of surveys, with 325 statements meeting the criteria for inclusion in the redeveloped guidelines. 59 panel participants completed all three surveys.

**Conclusions:**

The redeveloped guidelines outline a general set of strategies for providing initial assistance to a person who is experiencing psychosis. Compared to the original guidelines, these redeveloped guidelines provide more detailed instruction for members of the public on how to provide mental health first aid to assist a person experiencing psychosis. The guidelines are available to the public and will be used to update the Mental Health First Aid courses.

## Background

In 2018, the estimated global 12-month prevalence of psychotic illnesses was 0.40% [1]. While psychotic illnesses are less common than other mental illnesses, their effects can be debilitating and persistent, with people with a psychotic illness more likely than the general population to be unemployed, homeless, have lower educational attainment, and experience poorer physical health and global functioning [2].

Early intervention and treatment for psychosis is important, with delayed treatment for first episode psychosis associated with poorer clinical and functional outcomes [3]. There are, however, a range of factors that can contribute to delays in professional help-seeking for psychosis, including low mental health literacy, self-stigma, and perceived public stigma [4]. Conversely, the support of friends and family can facilitate help-seeking among people experiencing first-episode psychosis [4]. It is therefore important that members of the community have the knowledge, skills and confidence to recognise if a person may be experiencing psychosis, provide appropriate support and encourage help-seeking behaviours. This assistance is known as mental health first aid and is provided until professional help is received, or in the event of a mental health crisis (e.g. suicidality), until the crisis resolves [5]. However, public knowledge about psychosis is more limited than other mental health conditions such as depression, suicide, self-injury and substance misuse [6]. Furthermore, the quality of helping behaviours toward a person with psychosis is considerably lower than for other mental health conditions, highlighting the importance of public health interventions that provide knowledge and promote appropriate helping behaviours [7].

The Mental Health First Aid (MHFA) program was established in 2000 to teach members of the public mental health first aid skills [5]. The MHFA program has since spread to 24 countries and over 4 million people have been trained globally [8]. MHFA courses are based on guidelines developed through the Delphi method, a systematic way of determining expert consensus on a topic that is not amendable to experimental study designs [9]. The Delphi method has been used to develop standards of practice, policies, and guidelines on prevention and early intervention strategies. The guidelines for providing mental health first aid are distinct from clinical practice guidelines in that they are designed for people without mental health qualifications, such as family members, friends, colleagues, and concerned community members, with an aim of encouraging the person experiencing the mental health problem to seek appropriate professional help.

The Delphi method has been used to develop a range of mental health first aid guidelines, including guidelines for specific mental health problems such as suicidal ideation and behaviour, depression, non-suicidal self-injury and panic attacks, and specific population groups such as older people, Australian Aboriginal and Torres Strait Islander people and people from immigrant and refugee backgrounds [10]. There are existing mental health first aid guidelines for psychosis. These were developed in 2008 using the Delphi method [11]. As with clinical practice guidelines, mental health first aid guidelines are updated regularly to ensure they reflect current evidence. In accordance with MHFA International’s practice of updating guidelines every ten years, the current research project aimed to redevelop the existing mental health first aid guidelines for psychosis. As the guidelines are based on expertise gained through either personal or professional experience with psychosis, the study required the consensus of Carer, Consumer and Professional panels with expertise on the topic.

The aim of this Delphi expert consensus study aimed was to redevelop the mental health first aid guidelines on how a member of the public can recognise and respond to a person experiencing psychosis.

## Methods

### Delphi method

The Delphi method was used to redevelop the mental health first aid guidelines for psychosis, first developed in 2008. The study was conducted across five stages: (1) the formation of expert panels, (2) a systematic literature search, (3) questionnaire development, (4) data collection and analysis, (5) the redevelopment of the guidelines.

### Panel formation

The Delphi method is based on the premise of the ‘wisdom of crowds’, through which consensus is determined between groups or ‘panels’ of individuals with a diversity of expertise in the topic of interest [9]. The study aimed to recruit participants to three expert panels: Carer, Consumer and Professional. Participants were eligible if they were 18 years or over, could read and write in English, and fitted one or more of the criteria outlined in Table 1.

**Table 1:** Expert panel eligibility criteria

Expert panel	Criteria
Carer	Have experience in caring for or providing day-to-day support to someone with psychosis AND are engaged in activities that give you a broader exposure to people’s experiences of psychosis, e.g. are a member of a carer support group or carer advocacy organisation, etc
Consumer	Have a lived experience of psychosis, feel well enough to participate AND are engaged in activities that give you a broader exposure to people’s experiences of psychosis, e.g. are a member of a consumer advisory or advocacy group, providing peer support to others, etc
Professional	Are a mental health professional, educator or researcher with at least 5 years’ experience in the area of psychosis

Participants who fitted the criteria for more than one expert panel were asked to select the one they most identified with, which determined the expert panel they were assigned to.

Delphi studies often involve one expert panel of Professionals. However, the value of incorporating the views of Carers and Consumers has been recognised [9]. The three expert panels in the current study were established to include a diversity of expertise on the topic of mental health first aid for psychosis. While the 2008 Delphi study had Carer, Lived Experience, and Clinician panels, the current study expanded the Clinician panel to be a broader ‘Professional’ panel that included mental health professionals, educators and researchers.

As the purpose of the study was to develop guidelines suitable for high-income Western countries with developed health systems, participants were eligible if they were from relevant countries that had a MHFA program (Australia, Canada, Denmark, Finland, France, Germany, Ireland, The Netherlands, New Zealand, Sweden, Switzerland, United Kingdom, and the United States of America).

### Recruitment

Relevant organisations and individuals from each eligible country were targeted. A flyer advertising the study was distributed to mental health organisations and professional bodies (e.g. Mental Health Australia, Finnish Psychological Society, Schizophrenia Society of Canada, Mental Health Advocacy and Peer support New Zealand) requesting that they distribute it through their networks. Individuals with relevant expertise (e.g. researchers with published work in the field; publicly known consumers; authors) were identified through their professional or advocacy roles. Individuals were invited directly via email and were provided with a Plain Language Statement. Confirmed and potential participants were also asked to let others know about the research as a way of expanding the expert panels. All individuals and organisations contacted were advised that mental health professionals were not to recruit their patients or their patients’ families. The study was also advertised through MHFA Australia’s network of Instructors, newsletter, website and social media, and relevant organisations that have licensed the MHFA program internationally. MHFA courses are delivered by Accredited MHFA Instructors, who are often Carers, Consumers and Professionals, and they were eligible to participate. However, this was capped at 50 per cent of each panel to limit potential bias associated with familiarity with the original guidelines.

All individuals who expressed interest were provided with a Plain Language Statement and an individual link to the online survey. Participants were not reimbursed for participation.

To enable a meaningful consensus to be achieved, the study aimed to recruit a minimum of 30 participants to each panel, in accordance with the recommended minimum participants per panel at the start of a Delphi study [10]. A panel size of at 23 has been found to yield stable results when using strict inclusion criteria, with the recruitment target for this study allowing for anticipated attrition across the study [10, 12].

### Literature search

A systematic search of academic and grey literature (websites, brochures, fact sheets and training material) was undertaken over two consecutive days in April 2017 to identify knowledge and skills a first aider may need in order to provide mental health first aid to a person who may be experiencing psychosis. The knowledge and skills are known as “mental health first aid strategies” or “first aid strategies”.

The first author conducted the literature search using geo-targeted search engines (Google (USA; UK; AU); Google Books and Google Scholar), to identify contextually relevant first aid strategies. As academic articles rarely provide information on first aid strategies, Google Scholar was the only academic search engine used due to its broader interdisciplinary coverage. To minimise the influence of Google’s searching algorithm, the researcher signed out of any Google profiles, cleared their search history and used incognito mode.

The search was restricted to sources published post-June 2006, as this was the date that the 2008 psychosis Delphi study’s literature search was conducted and the purpose was to find more recently published strategies for inclusion in the study. The current study incorporated endorsed strategies from the original Delphi study and new strategies identified in the current literature search.

The search terms used across each search engine included the search terms that were used in the original 2008 study (“psychosis”, “first-episode psychosis”, “schizophrenia”, “help for psychosis”, “help for schizophrenia”, “psychosis family friends”, “schizophrenia family friends”, “psychosis carer”, “schizophrenia carer”, “help for hearing voices”, “help for hallucinations delusions”, “family + psychosis + violence”, “psychosis + doesn’t want help”) [11]. Additional search terms were also used. These included phrases that may be used by the general community (“how to help someone with psychosis”, “help for someone with schizophrenia”) and phrases incorporating the concept of mental health first aid (“first aid for psychosis”, “first aid for schizophrenia”), which have become more common since the original Delphi study was undertaken.

**Fig. 1:**
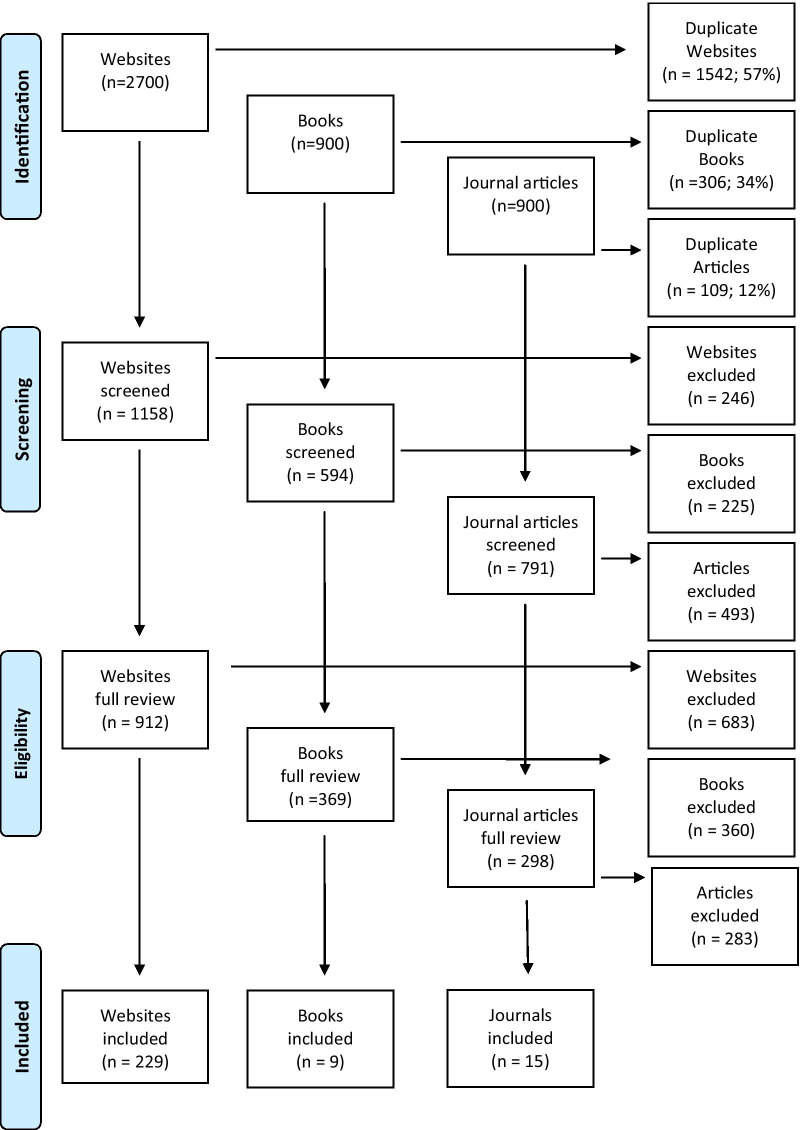
Summary of literature search

The first 50 results from each search engine and search term were retrieved. Sources were de-duplicated, with the remaining sources screened for relevance. Sources were excluded if they were newspapers, blogs, forums, or not related to both mental health first aid and psychosis (e.g. were about physical first aid or helpful strategies for another mental health problem). Any additional sources that were linked to from these websites were also reviewed. The combination of search engines, search terms, and the number of sources retrieved was based on unpublished observations and previous Delphi studies [13], which found that using additional search engines from more countries and retrieving more than the first 50 Google results did not provide any additional first aid strategies. The search strategy was found to be comprehensive enough to develop a wide range of first aid strategies for psychosis, as indicated by the high number of duplicate sources and the repetition of strategies across sources. See Fig. 1 for a summary of the literature search.

### Survey questionnaire development

The study involved three consecutive rounds of online survey questionnaires that contained statements about strategies for how a member of the public could provide mental health first aid to a person experiencing psychosis. The first author reviewed all sources retrieved from the literature search to identify knowledge and skills that a first aider may need in order to provide mental health first aid to a person experiencing psychosis. The role of the research team (comprising the authors of this paper) in reviewing the sources was to compile a list of clear and actionable statements to be rated by participants. The researchers did not make judgements about the content of the strategies, as these reflect the wide variety of beliefs about how to support someone with psychosis.

Strategies identified through the search were extracted ‘word-for-word’ and reviewed by the research team who drafted them into single-idea, action-oriented statements that maintained the original source’s meaning. These statements formed the Round 1 survey questionnaire. In addition to the statements developed from the literature search, all questionnaire statements that were endorsed in the original 2008 Delphi study were also included in the Round 1 survey questionnaire of the current study. Statements that were rated in the 2008 Delphi study, but not endorsed for inclusion in the original guidelines, were not included. The statements were sorted into themes (e.g. recognising and acknowledging the person may be experiencing psychosis; being supportive; hallucinations and delusions). Example questionnaire statements are shown in Table 2.

**Table 2:** Example questionnaire statements

Example of original strategies from literature search	Example questionnaire statements
Accept that the person is experiencing symptoms that are beyond his/her control	The first aider should know that the person is experiencing symptoms that are beyond their control and should not blame them or take their actions personally
Don’t ask him or her to try to force the voices to stop	The first aider should know that it is not helpful to encourage the person to try to stop hallucinations

### Data collection and analysis

Participants completed the three consecutive rounds of online questionnaires via Survey Monkey, in which they rated how important they thought each statement was to be included in the mental health first aid guidelines for psychosis (‘essential’, ‘important’, ‘don’t know/depends’, ‘unimportant’, ‘should not be included’). See Additional files 1, 2 and 3 for the three survey questionnaires.

The quantitative data was statistically analysed to measure the level of consensus across the expert panels, with statements categorised based on the following criteria:

- An statement was endorsed for inclusion in the guidelines if it received an ‘essential’ or ‘important’ rating from 80 to 100% of members from each panel.
- An statement required re-rating if it received an ‘essential’ or ‘important’ rating from 70 to 79% of members of each panel, or an ‘essential’ or ‘important’ rating from 70 to 79% of one panel and above 80% from the other panels.
- An statement was rejected if it was rated as ‘essential’ or ‘important’ by less than 70% of at least one panel.

The Round 1 survey questionnaire collected demographic information (age, gender, country) and asked participants to specify which expert panel they met the criteria for. Participants were asked to identify the relevant organisation they had worked or volunteered for, and their role within the organisation. Participants were asked if they were an Accredited MHFA Instructor so that this could be capped at 50 per cent of the participants of each panel. While confidentiality was protected, names and emails were collected so the researchers could identify which participants had completed each survey round and were therefore eligible to complete the following round. The Round 1 survey questionnaire also provided participants the opportunity to contribute additional first aid strategies that had not emerged in the literature search, through an open-ended question at the end of each section asking *‘Please provide any additional items or comments related to this section’.* The research team reviewed this qualitative data and developed new questionnaire statements based on novel ideas. In the Round 2 survey, participants were asked to re-rate any statements that were neither endorsed nor rejected in Round 1 and rate any new statements derived from the participant comments in Round 1. In the Round 3 survey, participants re-rated any statements that were neither endorsed nor rejected in the previous round.

After each of the Round 1 and Round 2 surveys participants were provided with a personalised report summarising the results of the respective survey. Each report comprised three sections. The first section included the statements that required re-rating, and provided tabulated summaries of the percentage ratings of each panel and the individual participant’s own ratings. This enabled participants to consider each expert panel’s ratings when re-rating statements in the next survey round. The second and third sections of the report included the endorsed statements for inclusion in the guidelines and the rejected statements, respectively.

### Guidelines development

The statements that were endorsed by the expert panels were incorporated into a draft guidelines document developed by the research team. The draft guidelines were emailed to participants who completed all three survey rounds so they could provide final comments and feedback. Participants were not able to provide suggestions for new content at this final stage, but could provide suggestions to improve the structure and clarity of the guidelines. The feedback was reviewed by the research team and incorporated into the final guidelines document where relevant.

## Results

### Participants

Fifty-nine participants completed all three surveys (see Table 3 for retention rates). Participant characteristics are presented in Table 4. In the Round 1 survey, participants identified their primary source of expertise, which determined the expert panel they were assigned to. Thirty-two participants who completed all three surveys identified their primary expertise as a professional, educator or researcher in the field of psychosis (assigned to Professionals panel). Seventeen identified as a person with a lived experienced of psychosis (assigned to Consumer panel). Ten identified as a person with experience caring for or providing day-to-day support to a person with psychosis (assigned to Carer panel). While the study initially aimed to recruit participants to three expert panels, the number of Carer participants and Consumer participants was below the minimum of 23 recommended per panel to achieve stable results [12]. In balancing the need for meaningful consensus to be achieved with minimising the risk of participant attrition by extending the recruitment period, the Carer and Consumer participants were combined into one ‘Lived Experience’ expert panel. This decision was informed by similar studies that were undertaken with one or two panels [14, 15]. The percent of participants endorsing each statement was found to be highly correlated across the Carer and Consumer panels (r = 0.80), showing that they made similar judgements and justifying pooling the panels. Furthermore, a number of participants had secondary expertise as a Carer (18) or Consumer (7). The primary and secondary expertise of participants is presented in Table 5.

**Table 3:** Participant retention rates across three survey rounds

Expert Panel	Survey 1	Survey 2	Survey 3	Retention rate (%)
Professional	46	36	33	72
Lived Experience	39	29	26	67
TOTAL	85	65	59	69

Lived Experience panel is based on Carer and Consumer participants combined

**Table 4:** Characteristics of participants who completed 3 surveys (data collected in Round 1)

	Professional (n = 32)	Consumer (n = 17)	Carer (n = 10)	TOTAL (n = 59)
Female	19	6	8	33
Male	13	10	2	25
Identify with another term	0	1	0	1
Age range	30–69	20–64	41–75	20–75
Median age	47	45	50	47
MHFA Instructors	11	1	1	13
Australia	16	9	6	31
Canada	5	3	2	10
Ireland	0	1	1	2
Netherlands	1	0	0	1
New Zealand	0	1	0	1
Sweden	1	0	0	1
Switzerland	1	0	0	1
UK	7	3	1	11
USA	1	0	0	1
Total	32	17	10	59

**Table 5:** Primary and secondary source of expertise of participants who completed all 3 survey rounds (data collected in Round 1)

	Professional	Consumer	Carer
Primary Expertise	32	17	10
Secondary Expertise—Professional	NA	9	3
Secondary Expertise—Consumer	4	NA	3
Secondary Expertise—Carer	12	6	NA
Total	48	32	16

### Statement ratings

**Fig. 2:**
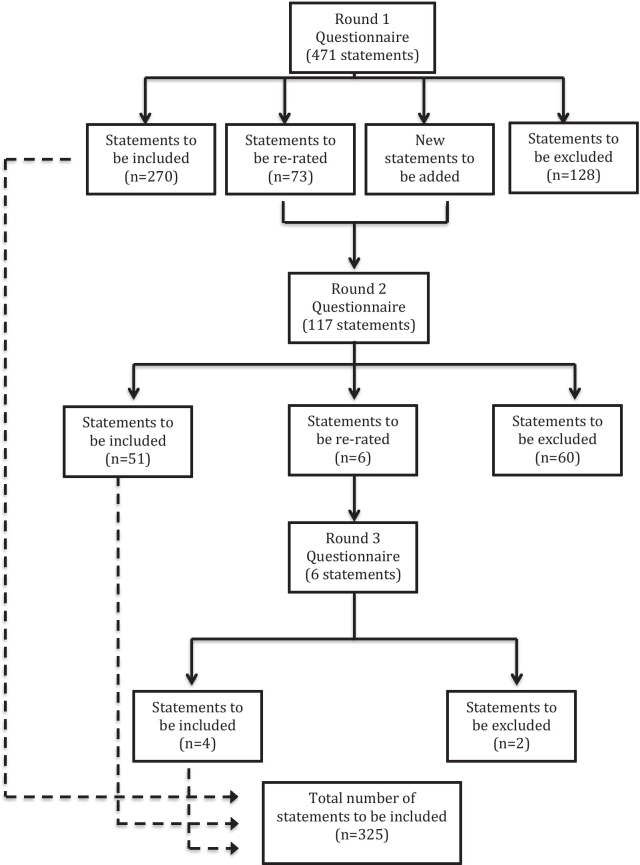
Summary of statement ratings

A total of 515 statements were rated over the three survey rounds. Three hundred and twenty-five statements were endorsed for inclusion in the guidelines and 190 were excluded. Figure 2 presents the number of statements included, excluded and re-rated across each survey round. A list of endorsed and rejected statements are presented in Additional file 4. The endorsed statements formed the basis of the revised guidelines for providing mental health first aid to a person who is experiencing psychosis.

Pearson’s r was calculated to determine the correlation across statements in endorsement rates between the Lived Experience and Professional panels. For the 471 statements rated in Round 1, the endorsement rates were strongly correlated with a correlation coefficient of 0.91 (*p* < 0.00001; t(47.57); df(469)). Correlations between the panels ratings for each of the sections were also calculated and presented in Table 6.

**Table 6:** Items endorsed and rejected and correlations by Delphi questionnaire sections

Topic	# endorsed	# rejected	Correlation between panel ratings (Pearson’s r)					
			*Round 1*			*Round 2*		
			*r*	*df*	*p*	*r*	*df*	*p*
Recognising and acknowledging that someone may be experiencing psychosis	16	2	.75	14	.000	–		
Knowing about psychosis	13	2	.35	11	.120	–		
When and how to approach the person	9	11	.96	18	.000	–		
Guidelines for good communication	8	9	1.0	7	.000	–		
Dealing with problems during the conversation	14	5	.95	17	.000	–		
Being supportive and understanding	18	6	.96	22	.000	–		
Treating the person with dignity and respect	9	2	.94	9	.000	–		
Encouraging professional help—general	6	6	.80	10	.001	–		
Supporting the person to seek professional help	6	8	.75	12	.001	–		
If the person doesn’t want professional help	10	2	.93	10	.000	–		
Responding to hallucinations and delusions	10	11	.96	19	.000	–		
Safety considerations when the person is in a severe psychotic state or behaving aggressively	27	5	.96	31	.000	–		
Communicating with the person when they are in a severe psychotic state or behaving aggressively	12	12	.97	22	.000	–		
De-escalation when the person is in a severe psychotic state or behaving aggressively	31	4	.39	33	.010			
Seeking help for the person when they are in a severe psychotic state or behaving aggressively	2	8	–			.41	8	.120
Calling emergency services for help when the person is in a severe psychotic state or behaving aggressively	18	0	.45	16	.030			

Correlations are not reported for sections where there were less than 10 items rated. Correlations for Round 3 have not been provided as there were not enough ratings to determine a valid correlation coefficient

### Similarities between the panel ratings

The 2019 panels generally agreed on statements relating to the scope of the first aider’s role, when and how to approach the person, guidelines for good communication, managing difficulties that arise during a discussion, recommendations for other supports, postnatal psychosis and self-care for the first aider. For example, both panels endorsed all statements relating to self-care for the first aider, with high endorsements rates (> 93%) across these statements. Similarly, all statements relating to postnatal psychosis were endorsed by both panels, indicating that professionals and people with lived experience generally recognise the need for immediate action and timely receipt of professional help in such circumstances.

There were also similarities between the two panels regarding the statements that they rejected. None of the statements providing instruction on what to do if the person does seek professional help were endorsed, e.g. *If the person agrees to seek professional help, the first aider should help them to write a list of questions or points that they want to discuss with their health professional*. This may reflect the views of the panels in regards to the scope of a first aider’s role, i.e. that providing support once the person decides to seek professional help is not within the scope of this role.

Statements that provided strategies for encouraging other supports, such as connecting the person with a role model, support groups, engaging in education and employment programs, and engaging in a healthy lifestyle, were rejected by both panels. Participant comments indicate that both panels considered the suitability of such recommendations to be dependent on the first aider’s relationship with and knowledge of the person, as well as the person’s own circumstances.

### Differences between the panel ratings

There were differences in the statement ratings between the expert panels, with a number of statements endorsed by one panel and rejected by the other panel. Six were endorsed by the Professional panel but rejected by the Lived Experience panel by ≥ 10%. Conversely, 18 were endorsed by the Lived Experience panel but rejected by the Professional panel by ≥ 10%. Statements that were rejected by one panel with a difference of 10% are presented in Additional file 4.

The statements that were rejected by the Lived Experience panel but endorsed by the Professional panel, with a margin of at least 10% included statements about discussing the person’s thoughts or behaviours, suggesting the person seek professional help and where the person should stand in relation to the person if they are in a severe psychotic state or behaving aggressively. The statements that were rejected by the Professional panel but endorsed by the Lived Experience panel with a margin of at least 10% included various statements about talking to the person or communication tips, two items around hallucinations or delusions, safety concerns when you are alone with the person and the person helping the person arrange or manage their professional appointments.

While both panels endorsed statements relating to professional help-seeking, there were differences in panel ratings. For example, the following statement was endorsed by the Professional panel but rejected by the Lived Experience panel:

- ‘The first aider should suggest to the person that they seek professional help.’

Both panels, however, endorsed a similar version that was included in the Round 2 survey:

- ‘The first aider should encourage the person to seek professional help.’

These results indicate that while both panels think it is important that the person seeks professional help, Professionals may consider a more direct approach of ‘suggesting’ professional help to be appropriate than people with Lived Experience do.

Other differences relating to professional help-seeking were primarily concerned with crisis situations. For example, the following statement was endorsed by the Lived Experience panel but not the Professional panel:

- ‘If the person is in a severe psychotic state and receiving professional help for psychosis, the first aider should contact the person’s health professional immediately.’

It may be that in such a situation, Professionals consider it more appropriate to call a crisis team or emergency services for immediate assistance than the person’s health professional, as indicated by a participant who stated: ‘Emergency service may be more appropriate’. It also appears that Professionals may prefer more direct intervention in a crisis situation, as demonstrated by the panel’s rejection of the following statement:

- ‘If the person needs to go to hospital, the first aider should encourage the person to go voluntarily.’

The Lived Experience panel was more likely to endorse strategies that are responsive to the person’s comfort and preferences, for example:

- ‘The first aider should not continue talking about a topic if it is distressing for the person.’

The Professional panel rejected this statement, indicating that a level of distress when talking to a person who has been experiencing psychosis is to be expected and is not a reason to discontinue a discussion. This is illustrated by participants who commented: ‘How much distress? A little bit is to be expected, so the first-aider needs to be prepared for this, otherwise the person may avoid the conversation altogether…’ and ‘Talking about difficult things may be distressing for them, but it may also be important for them to be able to voice their distress’.

The statements rejected by the Professional panel suggest professionals are generally less supportive of strategies that could increase the risk of harm to the person. This is illustrated by the rejection of the following statement by the Professional panel which was endorsed by the Lived Experience panel:

- ‘If the person is behaving aggressively, the first aider should leave the person alone until they have calmed down, if it is safe to do so.’

While this particular strategy is qualified by the inclusion of ‘if it is safe to do so’, a number of professionals indicated that giving the person space is preferable to leaving them alone, stating: ‘Give them space but don’t leave them’ and ‘If the first aider gives space to the person to calm down, the person should not be left alone’.

Both panels tended to agree about which strategies relating to aggressive behaviours should not be included in the guidelines. However, there was one exception that was endorsed by the Lived Experience panel but rejected by the Professional panel:

- ‘Until the first aider knows the content and context of the person’s delusions, it is important to keep themselves safe from potentially aggressive reactions.’

This statement assumes that the person may become aggressive, linking delusions to aggressive behaviours. This may have contributed to the Professional Panel rejecting this statement, as indicated by a participant who stated: ‘Sane Australia says “Violence is not a symptom of psychotic illnesses like schizophrenia. The causal link between psychosis and violence is inconclusive.” It is not fair to “look for” signs of violence in a person with psychosis or any mental illness…. violence should not be anticipated any more than it would be for any other person.’ Another Professional reinforced this view, stating that this statement ‘may serve to reinforce the myth that people with psychosis are dangerous ‘.

### Difference between the 2008 and 2019 Psychosis guidelines

A total of 325 statements were endorsed for inclusion in the 2019 guidelines, compared with 89 in the 2008 guidelines [11]. Minor changes to wording were made to improve the clarity of 43 statements. Forty-four items from the 2008 Delphi were re-endorsed, while 5 were rejected. The sections of the redeveloped guidelines compared with the sections of the 2008 guidelines are presented in Table 7. See Additional file 4 for a comparison of statements endorsed in 2019 with those in 2018. The sections of the redeveloped guidelines compared with the sections of the 2008 guidelines are presented in Table 8. See Additional file 4 for a comparison of statements endorsed in 2019 with those in 2018. Pearson’s r was calculated to determine the correlation between the endorsement rates across in 2008 and 2019 (see Table 8).

**Table 7:** Sections in the 2019 guidelines and the 2008 guidelines

Sections in 2019 Guidelines	Sections in 2008 Guidelines
What is psychosis?	
How do I know if someone may be developing psychosis?	How do I know if someone is experiencing psychosis?
Common signs and symptoms when psychosis is developing	Common symptoms when psychosis is developing
Things to avoid if you think a person may be experiencing psychosis	
How should I approach someone who may be experiencing psychosis?	How should I approach someone who may be experiencing psychotic symptoms?
How should I talk to the person about what they are experiencing?	
Tips for communicating with a person who may be experiencing psychosis Language Listening non-judgementally Body language	
How can I be supportive and understanding?	How can I be supportive?
How should I treat the person with dignity and respect?	
How should I respond to hallucinations and delusions? What not to do when responding to hallucinations and delusions	How do I deal with delusions and hallucinations?
What if the person is experiencing paranoia?	
What if the person’s communication is affected?	How do I deal with communication difficulties?
How do I respond to challenges during the discussion?	
Professional help: How should I encourage the person to seek professional help? How should I support the person to seek professional help? What if the person doesn’t want professional help?	Should I encourage the person to seek professional help? What if the person doesn’t want help?
What about self-help strategies and other supports?	
What if the person has recently given birth?	
What if the person has been using alcohol or other drugs?	
What if the person is in a severe psychotic state? Safety considerations when the person is in a severe psychotic state De-escalation when the person is in a severe psychotic state Seeking help when the person is in a severe psychotic state If you think the person is in a severe psychotic state and needs to go to hospital	What should I do in a crisis situation when the person has become acutely unwell?
What if the person appears to be behaving aggressively?	What if the person become aggressive? How to de-escalate the situation?
What if I think the person is at risk of suicide?	
How can I look after my own self-care?

**Table 8:** Correlation between endorsement rate between 2008 and 2019 Delphi studies

	Pearson’s r^b^	df	t
Lived Experience^a^	.48	39	.001
Professional	.32	39	.021

^a^The 2008 study had separate Carer and Consumer panels. Additional analyses were undertaken to combine the Carer and Consumer panels data into one Lived Experience panel

^b^In interpreting these correlations, it should be noted that only endorsed statements from the 2008 study were included in 2019, truncating the possible range of endorsement rates and attenuating correlations

Greater instruction on professional help-seeking is provided in the redeveloped guidelines, with a marked increase of 50 statements in the redeveloped guidelines compared with 14 in the original. This increase in the number of statements endorsed reflects the complexities of help-seeking, by recognising the various stages of help-seeking and clarifying the role of the first aider across these. For example, the original guidelines included two sections on professional help-seeking (*Should I encourage the person to seek professional help?* And *What if the person doesn’t want help).* The 2019 guidelines expand on this to include additional sections that provide guidance at different stages of the help-seeking pathway, including: *How should I support the person to seek professional help* and *Seeking help when the person is in a severe psychotic state.*

While the professional help sections in the redeveloped guidelines provide specific guidance at different stages of the help-seeking pathway, they also provide a greater depth of information. For example, there are three new statements relating to the knowledge a first aider should have about professional help and five new statements about providing the person with information and resources about professional help. Three statements were endorsed for inclusion in the new section: *If the person is in a severe psychotic state and needs to go to hospital.* This topic was not addressed in the original guidelines. As mental health first aid is provided until professional help is received, these additions provide more comprehensive guidance on how a person can support and facilitate professional help-seeking across all stages and extend the role of the first aider to situations in which a person may need to seek professional help in a hospital setting.

The redeveloped guidelines also differ to the original guidelines by recognising the need for first aiders to have a certain level of knowledge about psychosis in order to provide first aid. Thirteen such knowledge statements were endorsed in the redeveloped guidelines compared with two in the original guidelines, including the following statements:

- ‘The first aider should be aware that treatment is most effective when psychosis is detected early.’
- ‘The first aider should know that the person may be aware of what is happening to them, may have no insight at all, or may not accept that they are unwell.’

The complexity of recognising and acknowledging that a person may be experiencing psychosis is also addressed in greater detail in the redeveloped guidelines. Sixteen statements relating to this topic were endorsed for inclusion in the redeveloped guidelines, compared with seven in the original guidelines. In particular, the following statements that are new in the redeveloped guidelines recognise the complexity in attributing symptoms to psychosis:

- *‘*The first aider should be aware that a single sign or symptom does not necessarily indicate psychosis, but a group of signs or symptoms is more likely to.’
- ‘The first aider should know that even if the person exhibits signs and symptoms of psychosis, they do not necessarily have a psychotic illness.’

A number of new themes are also introduced in the redeveloped guidelines. Reflecting an increased understanding of the importance of self-care, the redeveloped guidelines now recognise the effect that providing first aid for psychosis may have on a first aider, and thus include a new section on self-care for the first aider. All statements relating to self-care for the first aider were endorsed for inclusion in the guidelines. While these statements were rated by participants specifically in relation to self-care in the provision of first aid for psychosis, the strategies may be relevant to the provision of first aid for other mental health problems.

The redeveloped guidelines also include a new section on postnatal psychosis, recognising the risks to mother and her baby. The urgency of seeking emergency medical assistance for new mothers experiencing psychosis is reflected in a number of statements including:

- ‘If the first aider thinks a mother may be experiencing postnatal psychosis, they should call a mental health crisis team immediately, as it can escalate rapidly and delays in treatment can lead to increased risk for the mother and her baby.’
- ‘If a mother has delusions that involve her baby, the first aider should call a mental health crisis team immediately.’

Self-help strategies and other supports for the person experiencing psychosis are addressed in considerably more detail in the redeveloped guidelines, with a new dedicated section providing guidance on this topic. This new section includes guidance on encouraging the person to try self-help strategies and to utilise the supports of friends and family if appropriate, with the endorsement of the following statements:

- The first aider should try to determine whether the person has a supportive social network and if they do, the first aider should encourage them to use these supports.’
- ‘The first aider should encourage the person to try self-help strategies, e.g. relaxation methods, physical activity, good sleep habits.’

The importance of language use is also addressed in the redeveloped guidelines, with related statements endorsed across a number of topics. This includes instruction relating to not using diagnostic terms to describe the person’s experience, for example the following two statements were both included in the language section of communication tips:

- *‘*The first aider should use the same terminology that the person uses to describe their experiences.’
- ‘The first aider should use everyday language (e.g. ‘stress’) to normalise the person’s experiences.’

The complexity of using diagnostic terms is also highlighted as the endorsement of the following two statements which are included in the section *Seeking help when the person is in a severe psychotic state*:

- ‘If the first aider contacts a mental health service, they should not label the person as ‘psychotic’, but rather outline any symptoms and immediate concerns.’
- ‘If the first aider calls emergency services they should explain that they are concerned the person may be experiencing psychosis.’

The endorsement of these statements demonstrates that appropriate use of language to describe the person’s experiences may differ based on who the first aider is talking to.

## Discussion

The Delphi method was used to redevelop the guidelines for providing mental health first aid to a person experiencing psychosis that were first developed in 2008. The guidelines form part of a suite of guidelines on providing mental health first aid for a range of mental health problems and population groups. Two expert panels reached consensus on the 325 statements that are included in the guidelines. They provide instruction on recognising if a person may be developing psychosis, approaching a person and discussing concerns, communication advice, how to be supportive, what to do if the person has been using alcohol or other drugs, considerations specific to postnatal psychosis, encouraging and supporting professional help, responding to hallucinations and delusions, what to do in a crisis situation (including aggressive behaviours), and self-care for the first aider. The guidelines are publicly available on the MHFA Australia website (mhfa.com.au) and will be used to update the content of MHFA courses in Australia and internationally.

### Comparisons between the 2019 Professional and Lived Experience panel ratings

The statement endorsement rates for the Professional and Lived Experience panels were strongly correlated, indicating that participants from both panels tended to agree on what information was considered important for inclusion in or exclusion from the guidelines. However, there were some broad areas of disagreement that may reflect their different roles and types of experience. For example, the Professionals preferred a more direct approach to assiting the person with appointments and crisis situations, where as the Lived Experience panel may have thought allowing the person more autonomy is preferrabe, a trend also observed in the 2008 Delphi [11].

### Comparison between the 2008 and 2019 guidelines

The number of statements endorsed for inclusion in the 2019 guidelines was considerably higher than that of the original guidelines (325 compared to 89) [11]. With 236 more statements endorsed for inclusion in the 2019 guidelines, the resulting guidelines include more detailed advice and the emergence of new topics.

A range of factors likely contributed to the higher number of endorsed statements and the emergence of new topics, including an updated definition of mental health first aid that incorporates the provision of assistance when a person is experiencing a worsening of an existing mental health problem, and expanded understanding of the scope of a first aider’s role. Furthermore, in the current study the literature search was undertaken separately across three geo-targeted search engines and utilised four additional search terms, yielding more sources from which statements were generated and a longer Round 1 survey questionnaire (471 statements compared to 146 in 2008) [11].

There were a range of similarities between the original and the redeveloped guidelines. Of the 89 statements endorsed in the original guidelines, 41 were re-endorsed in 2019 and 43 were re-endorsed with minimal changes to wording to improve clarity. This indicates that the majority of information in the original guidelines is still applicable in 2019. Five statements were not re-endorsed, however there were no clear patterns across these statements.

As outlined, there are a number of topics addressed in the redeveloped guidelines that were not addressed in the original guidelines. The topics of self-care for the first aider, postnatal psychosis and self-help and other supports for the person experiencing psychosis were not included in the surveys of the 2008 Delphi study and therefore participants were not required to rate them or invited to suggest first aid strategies on these topics. This may have been because the topics were considered out-of-scope and therefore any associated information was excluded from the study. Alternatively, the topics may have been in-scope, but no associated statements were generated from the literature review.

The redeveloped guidelines contain considerably more detailed instruction for a first aider than the original guidelines, a trend that is evident in the recent redevelopment of other guidelines [14, 16]. The increased level of information in the redeveloped guidelines reinforces the importance of updating guidelines on a regular basis.

### Strengths

A key strength of the study is that it resulted in redeveloped guidelines that reflect current literature and expert opinion on mental health first aid for psychosis. In doing so, the study identified a considerably greater number of first aid actions for inclusion in the guidelines, resulting in more direction on actions for a person providing mental health first aid for psychosis.

It is common for Delphi studies to involve one expert panel, usually professionals. This study involved three types of experts by also including Carers and Consumers. Furthermore, while participants of the original study included Carers, Consumers and Clinicians, the current study expanded the Clinician panel to be a broader ‘Professional’ panel that included professionals, researchers and educators. The expanded eligibility criteria allowed for a greater diversity of expertise to be represented, with such heterogeneity recommended for producing quality decisions [9]. Another strength is that the Delphi method enables panel members to respond to the surveys independently, ensuring that they are not influenced by other panel members [9].

Finally, although the majority were Australian, participants who completed the Round 1 survey were from 10 high-income Western countries with developed health systems, and those who completed all three surveys were from 9 of the countries. This was an increase from 6 countries represented in Round 1 of the original study [11] and thereby increases the generalisability of the guidelines to such settings.

### Limitations

The number of participants with primary expertise as a Carer or Consumer was lower than anticipated. However, a number of participants did have this form of secondary expertise. As discussed earlier, participants could draw on all of their expertise when responding to the surveys and therefore the representation of these two groups was higher than the primary expertise participation rates suggest.

The number of participants who completed all three survey rounds was lower in the current study than in the 2008 study, with longer surveys a likely contributor to participant attrition. Furthermore, experts may be more likely to contribute to a Delphi study that aims to develop new guidelines rather than redevelop existing guidelines. Despite this, the completion rates were higher than the minimum recommended size of 23 per panel [12].

As the expert panels were comprised of individuals with varying expertise in psychosis, participants may have been asked to rate statements that were outside their area of expertise. As a result, some important first aid actions may not have been endorsed for inclusion in the guidelines, due to experts rating them ‘don’t know/depends’. As participants couldn’t discuss their statement ratings with others, individual biases may not have been challenged. Furthermore, while the guidelines include adolescent-specific first aid actions, they do not incorporate cultural considerations. The guidelines are therefore suitable for providing first aid to adults and adolescents in high-income, Western countries with developed health systems and may not be suitable for other cultural groups and for countries with different health systems.

Finally, the redeveloped guidelines are considerably more detailed than the original guidelines. While this provides more guidance for first aid action, it may result in greater complexity in their implementation.

## Conclusion

The aim of this study was to undertake a Delphi study to update the mental health first aid guidelines for psychosis, first developed in 2008. The redeveloped guidelines outline a general set of strategies for providing initial assistance to a person who is experiencing psychosis, informed by current literature and the consensus views of people with expertise in psychosis (consumers, carers, researchers, professionals, and educators). The guidelines provide more detailed instruction for members of the public to undertake first aid for psychosis, compared to the original guidelines. The guidelines are available for download on the MHFA Australia website (mhfa.com.au) and will be used to inform the MHFA course curriculum in Australia and relevant countries with licensed MHFA programs.

## Supplementary Information

**Additional file 1.** Round 1 Survey.

**Additional file 2.** Round 2 Survey.

**Additional file 3.** Round 3 Survey.

**Additional file 4.** Endorsed and rejected statements.

## Data Availability

All data generated or analysed during this study are included in this published article as a additional file. The datasets analysed during the current study are available from the corresponding author on reasonable request.

## Abbreviation

MHFA: Mental health first aid
