# Supplementary material for: Offering mental health first aid to a person experiencing psychosis: a Delphi study to redevelop the guidelines published in 2008
Source: BMC Psychol. 2021 Feb 12;9:29. doi: 10.1186/s40359-021-00532-7 (PMC7881488; doi:10.1186/s40359-021-00532-7)
Supplement: Supplementary file 1 — Additional file 1. Round 1 Survey. [file 40359_2021_532_MOESM1_ESM.pdf]

Information about this research

Purpose of this research

Researchers from Mental Health First Aid Australia and The Centre for Mental Health at the University of Melbourne are collaborating to update the mental health first aid guidelines for psychosis. Mental Health First Aid Australia is a not-for-profit organisation focused on mental health training and research and The Centre for Mental Health is based at the Melbourne School of Population and Global Health at the University of Melbourne.

The aim of this current research project is to update the mental health first aid guidelines for how a member of the public should give assistance to a person who may be experiencing psychosis. These guidelines are being developed for high income Western countries.

There are current mental health first aid guidelines for psychosis that were developed in 2007/2008. Given that they are almost 10 years old, the present study aims to update these guidelines. The guidelines will be available for download on the Mental Health First Aid Australia website (mhfa.com.au) and will be used to inform the Mental Health First Aid course curriculum.

How we are doing it

The guidelines will be formed on the basis of expert consensus. We are using the consensus of mental health consumers and carers with advocacy/peer support experience and mental health professionals, educators and researchers. These experts will complete online surveys to provide their opinions on a range of strategies for how to help someone with psychosis. The strategies that receive a high level of endorsement will be included in the guidelines.

The strategies to be rated in the surveys are obtained from websites, books, fact sheets, brochures, scientific journal articles and training course materials. Some of the statements may seem contradictory or controversial. However, these are included because they reflect the wide range of people's beliefs about intervention and care.

What will you be asked to do?

You will be asked to complete three online surveys over about 4-6 months and the total estimated time commitment is approximately 2-3 hours.

Are there any risks?

We do not anticipate that you will experience any risk or discomfort by participating in this research.
In the unlikely event that you become distressed at some stage during your participation, you can contact the crisis support service in your country:

Australia: Lifeline on 13 11 14
Canada: National Suicide prevention Lifeline on 1800 273 TALK (8255)
Denmark: Suicide hotline 70 201 201
Finland: SOS Crisis Centre 010 195 202
France: Suicide Écoute 01 45 39 40 00
Germany: TelephoneSeelsorge 0800/111 0 111
The Netherlands: Suicide hotline 113Online
New Zealand: Lifeline Aotearoa on 0800 543 354
Republic of Ireland: Samaritans on 116 123
Sweden: Suicide hotline 020 22 00 60
Switzerland: PARSPAS 027 321 21 21
UK: Samaritans on 116 123
USA: National Suicide prevention Lifeline on 1800 273 TALK (8255)

If you change your mind

Participation in this project is voluntary. If you change your mind about participating, you are free to withdraw from the project at any time until the last survey is closed. You may also withdraw your data if you wish, up until the individual survey is closed. Simply contact the project coordinator, Fairlie Cottrill (fairliec@mhfa.com.au).

Your privacy

Any data we collect from you will be held under password protection and not divulged to others. We are interested in the consensus views of the panels, rather than the views of individual members, so your individual answers will never be reported. We will only present the results in statistical summary form. We occasionally use participant quotes in published journal articles. When this occurs we do not publish any identifying information with the quote.

Due to research code requirements, we will be storing the information collected for at least 5 years after the study, but none of your information will be identifiable or be able to be traced back to you.

If you have concerns about the project

This research project has been approved by the Human Research Ethics Committee of The University of Melbourne. If you have any concerns or complaints about the conduct of this research project, which you do not wish to discuss with the research team, you should contact the Manager, Human Research Ethics, Research Ethics and Integrity, University of Melbourne, VIC 3010. Tel: +61 3 8344 2073 or Email: HumanEthics-complaints@unimelb.edu.au. All complaints will be treated confidentially. In any correspondence please provide the name of the research team or the name or ethics ID number of the research project.

The ethics ID number of the research project is 1749330.1.

For more information

You received a Plain Language Statement when you expressed interest in this project. Please refer to this for more details about this study. The Plain Language Statement can also be accessed here.

Consent to participate

Do you meet the criteria to participate in this study?
You have been invited to participate in this research because you are 18 years or over,

AND

Have a lived experience of psychosis, feel well enough to participate, AND are engaged in activities that give you a broader exposure to people's experiences of psychosis, e.g. member of a consumer advisory or advocacy group, providing peer support to others, etc.

OR

Have experience in caring for or providing day-to-day support to someone with psychosis AND are engaged in activities that give you a broader exposure to people's experiences of psychosis, e.g. are a member of a carer support group or carer advocacy organisation, etc.

OR

Are a mental health professional, educator or researcher with at least 5 years experience in the area of psychosis.

* 1. Do you meet these criteria?

- ◯ Yes
- ◯ No, please exit the survey now.

Consent to participate

It is important for you to know that participation in this study is completely voluntary. You are not under any obligation to participate and you can withdraw at any time.

Best wishes,
The Mental Health First Aid Research Team

1. I consent to participate in this project, the details of which have been explained to me, and I have been provided with a written plain language statement to keep.

2. I understand that the purpose of this research is to update the Mental Health First Aid Guidelines for Psychosis.

3. I understand that my participation in this project is for research purposes only.

4. I acknowledge that the possible effects of participating in this research project have been explained to my satisfaction.

5. In this project I will be required to complete three online surveys over about 4-6 months.

6. I understand that my participation is voluntary and that I am free to withdraw from this project anytime without explanation or prejudice and to withdraw any unprocessed data that I have provided.

7. I understand that the data from this research will be stored at the Mental Health First Aid Australia and will be destroyed after 5 years

8. I have been informed that the confidentiality of the information I provide will be safeguarded subject to any legal requirements; my data will be password protected and accessible only by the named researchers.

9. I understand that given the small number of participants involved in the study, it may not be possible to guarantee my anonymity.

10. I understand that after I consent to participating, my data will be retained by the researcher.

* 2. I understand that by submitting this survey I am giving my consent to participate in this study.

- ○ Yes, I understand.
- ○ I do not consent to participating in this research. Please exit the survey now.

Instructions

Definitions used in this survey

Mental health first aid is the help offered to a person developing a mental health problem, experiencing a worsening of an existing mental health problem, or in a mental health crisis. The first aid is given until appropriate professional help is received or until the crisis resolves.

The person: the person who the mental health first aider is concerned may be experiencing psychosis.

The first aider: a concerned family member, friend, work colleague or member of the community, who provides help to a person who may be experiencing psychosis.

GP/Family doctor: a medical doctor based in the community who treats patients with minor or chronic illnesses and refers those with serious conditions to a specialist or hospital.

Professional/health professional: a broad range of health professionals through which a person may seek help for psychosis. This could include a mental health professional, GP/family doctor, or another health professional, e.g. allied health professional, hospital emergency staff.

Mental health professional: a health professional who is qualified to treat people who are experiencing psychosis, e.g. a psychologist, mental health nurse or psychiatrist.

Emergency services: services that respond to and deal with emergencies when they occur, e.g. emergency medical services (ambulance) or law enforcement (the police).

Mental health crisis service: services that respond to and provide immediate help during a mental health crisis and are responsible for assessing the care required by the person. Psychiatric nurses, social workers, psychiatrists and psychologists may work for a mental health crisis service.

Crisis: a person may be in a ***crisis*** associated with psychosis if:

- They are in a severe psychotic state, e.g. the person has overwhelming delusions and hallucinations, very disorganised thinking, or bizarre and disruptive behaviours. The person may appear very distressed, their behaviours may be disturbing to others, or they may behave in a way that endangers themselves or others.
- They appear to be showing aggressive behaviour. Aggressive behaviour can cause physical or emotional harm to others and may range from verbal abuse to physical abuse.
- They are experiencing suicidal thoughts or behaviours.

Instructions

Please complete the questionnaire by rating each statement according to how important you believe it is for inclusion in the guidelines for providing mental health first aid to a person who may be experiencing psychosis.

Please keep in mind that the guidelines will be used by the general public. **The statements need to be rated according to their importance for someone WITHOUT a counselling or clinical background.**

The majority of statements in the questionnaire pertain to both adults and adolescents. There are also a number of statements that clearly pertain *only to adolescents*. The adolescent-specific statements have been included as they are *additional considerations* that only apply to adolescents.

This questionnaire should take approximately 60-90 minutes to complete. You can complete the survey in two or more sittings. Your answers are saved when you click 'Next' at the bottom of a page. This marks your page and you can begin again at a later date on the next page. **Please be aware that once you have logged on and started responding you must complete the questionnaire on the same computer.**

In the next phase of the research you will be asked to complete another two surveys over approximately 6 months. The following two surveys will be considerably shorter and take less time to complete.

Overview of the questionnaire

Section 1: Recognising and acknowledging that someone may be experiencing psychosis
Section 2: Approaching the person
Section 3: Communication (non-crisis situation)
Section 4: Talking with the person (non-crisis situation)
Section 5: Communication difficulties
Section 6: Being supportive
Section 7: Substance use
Section 8: Postnatal psychosis
Section 9: Encouraging professional help (non-crisis situation)
Section 10: If the person doesn't want professional help (non-crisis situation)
Section 11: Hallucinations and delusions (non-crisis situation)
Section 12: Assessing whether the person is in crisis
Section 13: When the person is in crisis (severe psychotic state or behaving aggressively)
Section 14: Severe psychotic states (crisis situation)
Section 15: Aggression (crisis situation)
Section 16: Self-care for the first aider

Information about you

* 3. What is your name? (This allows us to determine who has completed the Round 1 survey and is therefore eligible to participate in Round 2. Your name will be deleted from your data when the project is complete).

* 4. How old are you?

* 5. What is your gender?

- Female
- Male
- I identify with another term
- Do not wish to disclose.

* 6. Please indicate your primary source of expertise, i.e. lived experience, carer or professional.

* 7. In addition to your primary source of expertise, do you also have experience of psychosis as a:

- Person with lived experience of psychosis
- Mental health carer or significant support person
- Mental health professional
- No other experience of psychosis

* 8. Please state the name of the organisation/s you work or volunteer for that make you eligible to participate in this study?

* 9. What is your role within the above organisation/s?

* 10. What country do you live in?

* 11. Are you a Mental Health First Aid Instructor, i.e. do you deliver the Mental Health First Aid course?

◯ Yes

◯ No

RECOGNISING AND ACKNOWLEDGING THAT SOMEONE MAY BE DEVELOPING PSYCHOSIS

This section contains statements about what the first aider needs to know about recognising and acknowledging that someone may be experiencing psychosis.

Please rate how important (from 'essential' to 'should not be included') you think it is that each statement be included in the guidelines.

Please keep our definitions in mind when responding to this section. You can access the definitions here.

There are **2 parts** to this section:

- Recognising and acknowledging that someone may be experiencing psychosis
- Knowing about psychosis

Recognising and acknowledging that someone may be experiencing psychosis

* 12. The first aider should be able to recognise the early signs and symptoms of psychosis.

- ○ Essential
- ○ Important
- ○ Don't know/depends
- ○ Unimportant
- ○ Should not be included

* 13. The first aider should be aware that changes that are very out of character for the person may be early signs of psychosis.

- ○ Essential
- ○ Important
- ○ Don't know/depends
- ○ Unimportant
- ○ Should not be included

* 14. The first aider should be aware that a single sign or symptom does not necessarily indicate psychosis, but a group of signs or symptoms is more likely to.

- Essential
- Important
- Don't know/depends
- Unimportant
- Should not be included

* 15. The first aider should know that even if the person exhibits signs and symptoms of psychosis, they do not necessarily have a psychotic illness.

- Essential
- Important
- Don't know/depends
- Unimportant
- Should not be included

* 16. The first aider should be aware that signs and symptoms of psychosis may vary from person to person and can change over time.

- Essential
- Important
- Don't know/depends
- Unimportant
- Should not be included

* 17. The first aider should be aware that the signs and symptoms of psychosis may appear suddenly or develop gradually over time.

- Essential
- Important
- Don't know/depends
- Unimportant
- Should not be included

* 18. The first aider should not ignore or dismiss signs and symptoms if they appear gradually or are unclear.

- Essential
- Important
- Don't know/depends
- Unimportant
- Should not be included

* 19. The first aider should not assume that any signs and symptoms of psychosis will go away on their own.

- Essential
- Important
- Don't know/depends
- Unimportant
- Should not be included

* 20. The first aider should be aware that symptoms of psychosis can be present in a number of mental illnesses.

- Essential
- Important
- Don't know/depends
- Unimportant
- Should not be included

* 21. The first aider should be aware that a person may experience psychosis as a single episode or as part of an ongoing illness, such as schizophrenia, bipolar disorder or dementia.

- Essential
- Important
- Don't know/depends
- Unimportant
- Should not be included

* 22. The first aider should be aware that the symptoms of psychosis may be related to drug or alcohol use.

- Essential
- Important
- Don't know/depends
- Unimportant
- Should not be included

* 23. The first aider should not assume that the person exhibiting signs and symptoms is 'just going through a phase', experiencing the normal ups and downs of life, or misusing substances.

- Essential
- Important
- Don't know/depends
- Unimportant
- Should not be included

RECOGNISING AND ACKNOWLEDGING THAT SOMEONE MAY BE DEVELOPING PSYCHOSIS Continued

Please rate how important (from ‘essential’ to ‘should not be included’) you think it is that each statement be included in the guidelines.

Please keep our definitions in mind when responding to this section. You can access the definitions here.

Recognising and acknowledging that someone may be experiencing psychosis - continued

* 24. The first aider should not dismiss changes in a young person's functioning or recurrent odd behaviour as being 'just a phase' or 'teenagers being teenagers'.

- Essential
- Important
- Don't know/depends
- Unimportant
- Should not be included

* 25. The first aider should not dismiss the person's lack of motivation or interest in life as laziness, as this may be a symptom of psychosis.

- Essential
- Important
- Don't know/depends
- Unimportant
- Should not be included

* 26. The first aider should take into consideration the spiritual and cultural context of the person’s behaviours.

- Essential
- Important
- Don't know/depends
- Unimportant
- Should not be included

* 27. If the first aider is unsure whether the person is experiencing psychosis, they should seek advice from a mental health professional.

- Essential
- Important
- Don't know/depends
- Unimportant
- Should not be included

* 28. If the first aider is unsure whether the person is experiencing psychosis, they should tactfully ask the person's friends or family whether the person has a diagnosis of psychosis or has experienced psychosis.

- Essential
- Important
- Don't know/depends
- Unimportant
- Should not be included

* 29. The first aider should ask the person's family or friends if they have noticed any concerning changes in their behaviour.

- Essential
- Important
- Don't know/depends
- Unimportant
- Should not be included

30. Please provide any additional items or comments related to this section.

RECOGNISING AND ACKNOWLEDGING THAT SOMEONE MAY BE DEVELOPING PSYCHOSIS (Continued)

Please rate how important (from 'essential' to 'should not be included') you think it is that each statement be included in the guidelines.

Please keep our definitions in mind when responding to this section. You can access the definitions here.

Knowing about psychosis

* 31. The first aider should try to learn more about psychosis, by seeking information from reputable online resources or mental health organisations.

- Essential
- Important
- Don't know/depends
- Unimportant
- Should not be included

* 32. The first aider should be aware that treatment is most effective when psychosis is detected early.

- Essential
- Important
- Don't know/depends
- Unimportant
- Should not be included

* 33. The first aider should be aware of the range of factors that may trigger psychosis, e.g. substance misuse, extreme stress or trauma.

- Essential
- Important
- Don't know/depends
- Unimportant
- Should not be included

* 34. The first aider should know that the person may be aware of what is happening to them, may have no insight at all, or may not accept that they are unwell.

- Essential
- Important
- Don't know/depends
- Unimportant
- Should not be included

* 35. The first aider should know that the person is experiencing symptoms that are beyond their control and should not blame them or take their actions personally.

- Essential
- Important
- Don't know/depends
- Unimportant
- Should not be included

* 36. The first aider should be aware that people experiencing psychosis or schizophrenia do not have 'split personalities'.

- Essential
- Important
- Don't know/depends
- Unimportant
- Should not be included

* 37. The first aider should be aware that psychosis is not contagious.

- Essential
- Important
- Don't know/depends
- Unimportant
- Should not be included

* 38. The first aider should be aware that psychosis is not an intellectual disability.

- Essential
- Important
- Don't know/depends
- Unimportant
- Should not be included

* 39. The first aider should be aware that psychosis can be very distressing and disruptive for**the person.**

- Essential
- Important
- Don't know/depends
- Unimportant
- Should not be included

* 40. The first aider should be aware that psychosis can be very distressing and disruptive for**people around the person.**

- Essential
- Important
- Don't know/depends
- Unimportant
- Should not be included

* 41. The first aider should be aware that psychosis can have a major impact on the person's everyday life, relationships, work or study.

- Essential
- Important
- Don't know/depends
- Unimportant
- Should not be included

* 42. The first aider should be aware that the person may experience multiple episodes of psychosis with periods of wellness in between.

- Essential
- Important
- Don't know/depends
- Unimportant
- Should not be included

* 43. The first aider should be aware that the person may lack social support because they isolate themselves or their behaviour leads others to withdraw from them.

- Essential
- Important
- Don't know/depends
- Unimportant
- Should not be included

* 44. The first aider should be aware that social support can be helpful for the person.

- Essential
- Important
- Don't know/depends
- Unimportant
- Should not be included

* 45. The first aider should be aware that they may feel a range of emotions (e.g. shock, confusion or guilt) when they realise someone close to them is experiencing symptoms of psychosis, and that these are common reactions.

- Essential
- Important
- Don't know/depends
- Unimportant
- Should not be included

46. Please provide any additional items or comments related to this section.

APPROACHING THE PERSON

This section contains statements about what the first aider needs to know about when and how to approach the person about the concerns they have for them.

Please rate how important (from ‘essential’ to ‘should not be included’) you think it is that each statement be included in the guidelines.

Please keep our definitions in mind when responding to this section. You can access the definitions here.

There is 1 part to this section.

When and how to approach the person

* 47. Where possible, first aider should prepare what they are going to say before approaching the person, e.g. by preparing two or three questions to ask the person.

- Essential
- Important
- Don't know/depends
- Unimportant
- Should not be included

* 48. The first aider should have resources with them that they can share with the person.

- Essential
- Important
- Don't know/depends
- Unimportant
- Should not be included

* 49. The first aider should be aware that a person developing psychosis may not reach out for help.

- Essential
- Important
- Don't know/depends
- Unimportant
- Should not be included

* 50. The first aider should approach the person rather than waiting to see if they reach out for help.

- Essential
- Important
- Don't know/depends
- Unimportant
- Should not be included

* 51. The first aider should let the person know that they would like to help and then wait for the person to approach them.

- Essential
- Important
- Don't know/depends
- Unimportant
- Should not be included

* 52. If the first aider does not know the person very well, they should not let this prevent them from approaching the person about their concerns.

- Essential
- Important
- Don't know/depends
- Unimportant
- Should not be included

* 53. The first aider should try to initiate a conversation with the person about their concerns as quickly as possible.

- Essential
- Important
- Don't know/depends
- Unimportant
- Should not be included

* 54. The first aider should approach the person face-to-face, if possible.

- Essential
- Important
- Don't know/depends
- Unimportant
- Should not be included

* 55. The first aider should avoid approaching the person using methods of communication that could lead to misunderstandings, (e.g. text, email, social media), unless there is no other option.

- Essential
- Important
- Don't know/depends
- Unimportant
- Should not be included

* 56. The first aider should approach the person for a one-on-one conversation, rather than a group discussion.

- Essential
- Important
- Don't know/depends
- Unimportant
- Should not be included

* 57. The first aider should try to approach the person in an environment that is likely to be safe, comforting and free of distractions.

- Essential
- Important
- Don't know/depends
- Unimportant
- Should not be included

* 58. The first aider should ask the person where they would like to meet.

- ◯ Essential
- ◯ Important
- ◯ Don't know/depends
- ◯ Unimportant
- ◯ Should not be included

APPROACHING THE PERSON Continued

Please rate how important (from ‘essential’ to ‘should not be included’) you think it is that each statement be included in the guidelines.

Please keep our definitions in mind when responding to this section. You can access the definitions here.

When and how to approach the person - continued

* 59. If the first aider cannot find the ideal time and place to talk to the person, they should still approach the person about their concerns rather than delaying the conversation.

- Essential
- Important
- Don't know/depends
- Unimportant
- Should not be included

* 60. The first aider should allow adequate time to have a conversation with the person, so that it is not rushed.

- Essential
- Important
- Don't know/depends
- Unimportant
- Should not be included

* 61. If the person wants to talk to the first aider but the first aider does not have time to, the first aider should tell the person this and offer to meet them another time when they can give them their full attention.

- Essential
- Important
- Don't know/depends
- Unimportant
- Should not be included

* 62. The first aider should try to be calm when approaching the person, regardless of the person's emotional state.

- Essential
- Important
- Don't know/depends
- Unimportant
- Should not be included

* 63. The first aider should try to find a time to talk to the person when both they and the person are calm.

- Essential
- Important
- Don't know/depends
- Unimportant
- Should not be included

* 64. The first aider should approach the person when they feel the person is most likely to be receptive.

- Essential
- Important
- Don't know/depends
- Unimportant
- Should not be included

* 65. The first aider should approach the person when they feel the person is most likely to be cooperative.

- Essential
- Important
- Don't know/depends
- Unimportant
- Should not be included

* 66. The first aider should approach the person in a caring and non-judgmental manner.

- Essential
- Important
- Don't know/depends
- Unimportant
- Should not be included

* 67. The first aider should tailor their approach and interaction to the way the person is behaving, e.g. if the person is suspicious and is avoiding eye contact, the first aider should be sensitive to this and give the person the space they need.

- Essential
- Important
- Don't know/depends
- Unimportant
- Should not be included

* 68. The first aider should not use the term ‘psychosis’ but rather discuss any concerning changes in thoughts, feelings or behaviour that they have noticed in the person.

- Essential
- Important
- Don't know/depends
- Unimportant
- Should not be included

* 69. The first aider should **not:**

	Essential	Important	Don't know/depends	Unimportant	Should not be included
...approach the person when they are feeling frustrated by the person's behaviour.	◯	◯	◯	◯	◯
...approach the person to discuss their concerns when the person is in a distracted, agitated	◯	◯	◯	◯	◯
...approach the person in a confrontational manner.	◯	◯	◯	◯	◯

70. Please provide any additional items or comments related to this section.

COMMUNICATION (in a non-crisis situation)

This section contains statements about what the first aider needs to know and do when communicating with a person who may be experiencing psychosis. This section is NOT about when the person is in crisis; later sections cover communicating during a crisis.

Please rate how important (from ‘essential’ to ‘should not be included’) you think it is that each statement be included in the guidelines.

Please keep our definitions in mind when responding to this section. You can access the definitions here.

There are **3** parts to this section:

- Guidelines for good communication
- Body language considerations
- Listening non-judgmentally

Guidelines for good communication

* 71. The first aider should convey empathy when communicating with the person.

- Essential
- Important
- Don't know/depends
- Unimportant
- Should not be included

* 72. The first aider should ask the person simple and direct questions.

- Essential
- Important
- Don't know/depends
- Unimportant
- Should not be included

* 73. The first aider should ask the person open-ended questions.

- Essential
- Important
- Don't know/depends
- Unimportant
- Should not be included

* 74. The first aider should use everyday language (e.g. 'stress') to normalise the person's experiences.

- Essential
- Important
- Don't know/depends
- Unimportant
- Should not be included

* 75. The first aider should avoid using psychiatric terms when talking to the person.

- Essential
- Important
- Don't know/depends
- Unimportant
- Should not be included

* 76. The first aider should try to avoid using stigmatising terms that may make the person feel defensive, e.g. crazy, nuts, psycho.

- Essential
- Important
- Don't know/depends
- Unimportant
- Should not be included

* 77. As far as possible, the first aider should let the person set the pace and style of the interaction.

- ◯ Essential
- ◯ Important
- ◯ Don't know/depends
- ◯ Unimportant
- ◯ Should not be included

78. Please provide any additional items or comments related to this section.

COMMUNICATION (in a non-crisis situation) Continued

Please rate how important (from ‘essential’ to ‘should not be included’) you think it is that each statement be included in the guidelines.

Please keep our definitions in mind when responding to this section. You can access the definitions here.

Body language considerations

* 79. The first aider should try to minimise body language that show distress or nervous behaviour, e.g. jiggling legs, fidgeting or nail biting.

- Essential
- Important
- Don't know/depends
- Unimportant
- Should not be included

* 80. The first aider should lean toward the person to show interest when talking to them.

- Essential
- Important
- Don't know/depends
- Unimportant
- Should not be included

* 81. The first aider should maintain eye contact when talking to the person, without staring at them.

- Essential
- Important
- Don't know/depends
- Unimportant
- Should not be included

* 82. The first aider should face the person squarely when talking to them.

- Essential
- Important
- Don't know/depends
- Unimportant
- Should not be included

* 83. The first aider should not touch the person without their permission.

- Essential
- Important
- Don't know/depends
- Unimportant
- Should not be included

* 84. If the person is sitting down, the first aider should not stand over them or hover near them.

- Essential
- Important
- Don't know/depends
- Unimportant
- Should not be included

85. Please provide any additional items or comments related to this section.

COMMUNICATION (in a non-crisis situation) Continued

Please rate how important (from ‘essential’ to ‘should not be included’) you think it is that each statement be included in the guidelines.

Please keep our definitions in mind when responding to this section. You can access the definitions here.

Listening non-judgmentally

* 86. The first aider should listen to the person non-judgmentally.

- Essential
- Important
- Don't know/depends
- Unimportant
- Should not be included

* 87. The first aider should listen carefully to the person and reflect what they hear so the person knows that they are listening.

- Essential
- Important
- Don't know/depends
- Unimportant
- Should not be included

* 88. The first aider should recap what the person has said in different words to check that they have understood correctly.

- Essential
- Important
- Don't know/depends
- Unimportant
- Should not be included

* 89. The first aider should ask the person clarifying questions to show that they are listening.

- Essential
- Important
- Don't know/depends
- Unimportant
- Should not be included

* 90. The first aider should show the person they are listening by saying things such as, "okay" and "I see".

- Essential
- Important
- Don't know/depends
- Unimportant
- Should not be included

* 91. The first aider should acknowledge what the person is saying and how they are feeling, e.g. "that sounds really upsetting" or "it sounds like you don't know what to do".

- Essential
- Important
- Don't know/depends
- Unimportant
- Should not be included

92. Please provide any additional items or comments related to this section.

TALKING WITH THE PERSON (in a non-crisis situation)

This section contains statements about what the first aider needs to know about talking with the person about their symptoms and dealing with problems that may arise in the discussion. This section is NOT about when the person is in crisis; later sections cover talking with a person during a crisis.

Please rate how important (from ‘essential’ to ‘should not be included’) you think it is that each statement be included in the guidelines.

Please keep our definitions in mind when responding to this section. You can access the definitions here.

There are 2 parts to this section:

- Talking to the person about their symptoms
- Dealing with problems during the discussion

Talking to the person about their symptoms

* 93. The first aider should try to find some common ground for discussion, gradually building up towards more specific questions about what the person is experiencing.

- ◯ Essential
- ◯ Important
- ◯ Don't know/depends
- ◯ Unimportant
- ◯ Should not be included

* 94. The first aider should not speculate to the person about a diagnosis.

- ◯ Essential
- ◯ Important
- ◯ Don't know/depends
- ◯ Unimportant
- ◯ Should not be included

* 95. The first aider should tell the person that they have noticed the person seems to be going through a tough time.

- Essential
- Important
- Don't know/depends
- Unimportant
- Should not be included

* 96. The first aider should focus on symptoms that the person is likely to feel more comfortable talking about, e.g. sleep problems rather than hearing voices.

- Essential
- Important
- Don't know/depends
- Unimportant
- Should not be included

* 97. The first aider should make gentle enquiries about any experiences the person is having that may be affecting their behaviour, e.g. hearing voices, unusual beliefs.

- Essential
- Important
- Don't know/depends
- Unimportant
- Should not be included

* 98. The first aider should ask the person to explain what they are experiencing in order to gain an understanding of the person's experiences from their own perspective.

- Essential
- Important
- Don't know/depends
- Unimportant
- Should not be included

* 99. The first aider should allow the person to talk about their experiences, feelings and beliefs if they want to.

- Essential
- Important
- Don't know/depends
- Unimportant
- Should not be included

* 100. The first aider should ask the person whether they have noticed changes in their own behaviour.

- Essential
- Important
- Don't know/depends
- Unimportant
- Should not be included

* 101. If the person has noticed changes in their behaviour, the first aider should ask whether they are distressed by these changes.

- Essential
- Important
- Don't know/depends
- Unimportant
- Should not be included

* 102. The first aider should ask the person how long they have been experiencing symptoms.

- Essential
- Important
- Don't know/depends
- Unimportant
- Should not be included

TALKING WITH THE PERSON (in a non-crisis situation) Continued

Please rate how important (from ‘essential’ to ‘should not be included’) you think it is that each statement be included in the guidelines.

Please keep our definitions in mind when responding to this section. You can access the definitions here.

Talking to the person about their symptoms - continued

* 103. The first aider should be aware that the person may be vague when describing their symptoms, and may emphasise physical symptoms over symptoms of mental illness.

- Essential
- Important
- Don't know/depends
- Unimportant
- Should not be included

* 104. The first aider should ask the person if they want to talk about how they are feeling.

- Essential
- Important
- Don't know/depends
- Unimportant
- Should not be included

* 105. The first aider should not probe or investigate details of the person's psychotic symptoms, e.g. by asking questions such as "what happens when you hear voices?" or "what do the voices say to you?".

- Essential
- Important
- Don't know/depends
- Unimportant
- Should not be included

* 106. The first aider should present their concerns to the person as their own opinion by using 'I' statements, e.g. "I have noticed…" or "I feel that…".

- Essential
- Important
- Don't know/depends
- Unimportant
- Should not be included

* 107. The first aider should state, using specific behavioural examples, why they are concerned about the person, e.g. "the other day you looked frightened and were saying things that I didn't understand."

- Essential
- Important
- Don't know/depends
- Unimportant
- Should not be included

* 108. The first aider should focus on just one of their concerns, rather than trying to discuss everything at once.

- Essential
- Important
- Don't know/depends
- Unimportant
- Should not be included

* 109. If the person is not paranoid, the first aider should ask a group of the person's trusted friends or family to talk to the person, as this can give a sense of agreement and concern, and minimise the chance that the person will turn on the first aider.

- Essential
- Important
- Don't know/depends
- Unimportant
- Should not be included

* 110. If the first aider is aware that the person has been prescribed medication for psychosis, they should check whether they are still taking it.

- Essential
- Important
- Don't know/depends
- Unimportant
- Should not be included

* 111. If the first aider finds out the person is not taking their prescribed medication they should encourage them to do so.

- Essential
- Important
- Don't know/depends
- Unimportant
- Should not be included

* 112. If the person is having trouble making decisions, the first aider should limit the number of options they offer the person.

- Essential
- Important
- Don't know/depends
- Unimportant
- Should not be included

113. Please provide any additional items or comments related to this section.

TALKING WITH THE PERSON (in a non-crisis situation) Continued

Please rate how important (from ‘essential’ to ‘should not be included’) you think it is that each statement be included in the guidelines.

Please keep our definitions in mind when responding to this section. You can access the definitions here.

Dealing with problems during the discussion

* 114. The first aider should recognise that even if the person does realise they are unwell, their confusion and fear about what is happening to them may lead them to deny that there is anything wrong.

- Essential
- Important
- Don't know/depends
- Unimportant
- Should not be included

* 115. The first aider should be aware that several conversations may be necessary before the person is open to talking.

- Essential
- Important
- Don't know/depends
- Unimportant
- Should not be included

* 116. The first aider should be aware that the person may be frightened by their thoughts and feelings.

- Essential
- Important
- Don't know/depends
- Unimportant
- Should not be included

* 117. The first aider should be aware that the person may react with emotions which don't seem to fit the context of the conversation, e.g. laughing.

- Essential
- Important
- Don't know/depends
- Unimportant
- Should not be included

* 118. If the conversation with the person becomes stressful or emotionally charged, the first aider should take a break to allow them and the person to calm down.

- Essential
- Important
- Don't know/depends
- Unimportant
- Should not be included

* 119. If the person is distressed by their experiences, the first aider should ask the person what will help them to feel safe or in control.

- Essential
- Important
- Don't know/depends
- Unimportant
- Should not be included

* 120. The first aider should avoid being negative or pessimistic when talking to the person about their future.

- Essential
- Important
- Don't know/depends
- Unimportant
- Should not be included

* 121. The first aider should not continue talking about a topic if it is distressing for the person.

- ◯ Essential
- ◯ Important
- ◯ Don't know/depends
- ◯ Unimportant
- ◯ Should not be included

* 122. If the person denies anything is wrong or does not wish to talk about what they are experiencing, the first aider **should:**

	Essential	Important	Don't know/depends	Unimportant	Should not be included
…point out changes that they have noticed in the person's behaviour	◯	◯	◯	◯	◯
...focus on listening rather than trying to change the person's mind.	◯	◯	◯	◯	◯
...ask the person if there is anything specific they can do to help them.	◯	◯	◯	◯	◯
...continue trying to have a conversation with the person until the person makes it clear that they do not want to talk.	◯	◯	◯	◯	◯
...let them know that they will be available to talk in the future.	◯	◯	◯	◯	◯

* 123. If the person denies anything is wrong or does not wish to talk about what they are experiencing, the first aider **should not:**

	Essential	Important	Don't know/depends	Unimportant	Should not be included
...argue with the person.	◯	◯	◯	◯	◯
...insist that the person is unwell.	◯	◯	◯	◯	◯
...try to force them to talk.	◯	◯	◯	◯	◯

* 124. If the first aider finds the person's behaviour annoying or irritating, they **should:**

	Essential	Important	Don't know/depends	Unimportant	Should not be included
...understand that the situation may be mutually distressing.	○	○	○	○	○
...let the person know how they are being affected.	○	○	○	○	○
...let the person know without using aggressive language.	○	○	○	○	○

125. Please provide any additional items or comments related to this section.

COMMUNICATION DIFFICULTIES

This section contains statements about what the first aider needs to know about communication difficulties that may occur and how to talk with a person who is experiencing communication difficulties.

Please rate how important (from ‘essential’ to ‘should not be included’) you think it is that each statement be included in the guidelines.

Please keep our definitions in mind when responding to this section. You can access the definitions here.

There are 2 parts to this section:

- Communication difficulties
- Helping the person to communicate

Communication difficulties

* 126. The first aider should be aware that the person may not be able to communicate in the way they normally would, e.g. responding with unrelated answers, or drifting from one topic to another.

- ◯ Essential
- ◯ Important
- ◯ Don't know/depends
- ◯ Unimportant
- ◯ Should not be included

* 127. The first aider should be aware that the person may miss nonverbal cues such as facial expression and tone of voice.

- ◯ Essential
- ◯ Important
- ◯ Don't know/depends
- ◯ Unimportant
- ◯ Should not be included

* 128. The first aider should not assume that the person cannot understand what they are saying, even if the person’s response is limited.

- Essential
- Important
- Don't know/depends
- Unimportant
- Should not be included

* 129. As the person may have difficulty following a conversation, the first aider should get to the point as quickly as possible.

- Essential
- Important
- Don't know/depends
- Unimportant
- Should not be included

* 130. If the person is showing a limited range of feelings, the first aider should be aware that it does not mean that this is all they are feeling.

- Essential
- Important
- Don't know/depends
- Unimportant
- Should not be included

* 131. The first aider should be aware that the person may be hearing voices, making it difficult for them to communicate.

- Essential
- Important
- Don't know/depends
- Unimportant
- Should not be included

* 132. If the person is behaving in an unusual way, the first aider should not draw attention to this or ask them about it.

- Essential
- Important
- Don't know/depends
- Unimportant
- Should not be included

* 133. If the person is having trouble communicating, the first aider should know that their presence alone can be reassuring for the person.

- Essential
- Important
- Don't know/depends
- Unimportant
- Should not be included

* 134. The first aider should avoid using complex language, e.g. metaphors or sarcasm.

- Essential
- Important
- Don't know/depends
- Unimportant
- Should not be included

* 135. The first aider should avoid strong displays of emotion, both positive and negative.

- Essential
- Important
- Don't know/depends
- Unimportant
- Should not be included

136. Please provide any additional items or comments related to this section.

COMMUNICATION DIFFICULTIES Continued

Please rate how important (from ‘essential’ to ‘should not be included’) you think it is that each statement be included in the guidelines.

Please keep our definitions in mind when responding to this section. You can access the definitions here.

Helping the person to communicate

* 137. The first aider should allow the person enough time to respond to questions or statements, as they may have difficulty processing information.

- ◯ Essential
- ◯ Important
- ◯ Don't know/depends
- ◯ Unimportant
- ◯ Should not be included

* 138. If the person is having trouble with spoken communication, the first aider should try to communicate through writing.

- ◯ Essential
- ◯ Important
- ◯ Don't know/depends
- ◯ Unimportant
- ◯ Should not be included

* 139. If the person is distressed but has trouble expressing their feelings, the first aider should suggest words that might describe how the person is feeling.

- ◯ Essential
- ◯ Important
- ◯ Don't know/depends
- ◯ Unimportant
- ◯ Should not be included

* 140. The first aider should try to communicate clearly and simply and repeat things where necessary.

- Essential
- Important
- Don't know/depends
- Unimportant
- Should not be included

* 141. If the person's speech has become disorganised, the first aider should focus on an element of the conversation that makes sense.

- Essential
- Important
- Don't know/depends
- Unimportant
- Should not be included

* 142. If appropriate and feasible, the first aider should check with others who know the person for advice on the best way to communicate with them.

- Essential
- Important
- Don't know/depends
- Unimportant
- Should not be included

143. Please provide any additional items or comments related to this section.

BEING SUPPORTIVE

This section contains statements about what the first aider needs to know about being supportive to a person who may be experiencing psychosis.

Please rate how important (from 'essential' to 'should not be included') you think it is that each statement be included in the guidelines.

Please keep our definitions in mind when responding to this section. You can access the definitions here.

There are 3 parts to this section:

- Being supportive and understanding
- Treating the person with dignity and respect
- Encouraging other supports

Being supportive and understanding

* 144. The first aider should reassure the person that they will be there to provide support.

- Essential
- Important
- Don't know/depends
- Unimportant
- Should not be included

* 145. The first aider should ask the person if, and how, they would like the first aider to support them.

- Essential
- Important
- Don't know/depends
- Unimportant
- Should not be included

* 146. The first aider should make it clear to the person what they are willing and able to do to support them.

- Essential
- Important
- Don't know/depends
- Unimportant
- Should not be included

* 147. The first aider should ask the person whether they would like practical support, e.g. arranging childcare for them or getting them to medical appointments.

- Essential
- Important
- Don't know/depends
- Unimportant
- Should not be included

* 148. If appropriate to the relationship, the first aider should:

	Essential	Important	Don't know/depends	Unimportant	Should not be included
...maintain their usual interactions with the person, e.g. by involving them in social events.					
...continue to reach out to the person, e.g. to let the person know they are thinking about them and that they care.					
…ask the person if it is ok to check in with them from time to time.					
...encourage the person to look after their physical health, e.g. by maintaining a healthy lifestyle and regular medical check-ups.					

* 149. The first aider should ask the person if there are any current stressors that may be contributing to their symptoms.

- Essential
- Important
- Don't know/depends
- Unimportant
- Should not be included

* 150. If the first aider has ongoing contact with the person, they should watch for signs that indicate the person may be experiencing a worsening of their symptoms.

- Essential
- Important
- Don't know/depends
- Unimportant
- Should not be included

* 151. In their ongoing interactions with the person, the first aider should not focus only on the person's mental health problems.

- Essential
- Important
- Don't know/depends
- Unimportant
- Should not be included

* 152. The first aider should accept that the person may not follow any suggestions the first aider makes.

- Essential
- Important
- Don't know/depends
- Unimportant
- Should not be included

* 153. If the person is distressed by their symptoms, the first aider should try to comfort them.

- Essential
- Important
- Don't know/depends
- Unimportant
- Should not be included

* 154. If the person is very fearful, the first aider should keep them company to reassure them that they are not alone.

- Essential
- Important
- Don't know/depends
- Unimportant
- Should not be included

BEING SUPPORTIVE Continued

Please rate how important (from ‘essential’ to ‘should not be included’) you think it is that each statement be included in the guidelines.

Please keep our definitions in mind when responding to this section. You can access the definitions here.

Being supportive and understanding - continued

* 155. The first aider should not try to immediately provide the person with solutions.

- Essential
- Important
- Don't know/depends
- Unimportant
- Should not be included

* 156. The first aider should not threaten consequences in an attempt to change the person's behaviour.

- Essential
- Important
- Don't know/depends
- Unimportant
- Should not be included

* 157. The first aider should reassure the person that they are there to help the person and want to keep them safe.

- Essential
- Important
- Don't know/depends
- Unimportant
- Should not be included

* 158. If the first aider has had personal experience with psychosis, whether in themselves or others, they should let the person know, as it can be reassuring.

- Essential
- Important
- Don't know/depends
- Unimportant
- Should not be included

* 159. The first aider should tell the person that they understand that the person may be frightened by what they are experiencing.

- Essential
- Important
- Don't know/depends
- Unimportant
- Should not be included

* 160. The first aider should not tell the person that they understand completely what the person is experiencing.

- Essential
- Important
- Don't know/depends
- Unimportant
- Should not be included

* 161. The first aider should not tell the person to get their act together.

- Essential
- Important
- Don't know/depends
- Unimportant
- Should not be included

* 162. The first aider should not interrupt the person with a story about themselves, even if it's relevant.

- Essential
- Important
- Don't know/depends
- Unimportant
- Should not be included

* 163. The first aider should discuss the changes they have observed with those close to the person.

- Essential
- Important
- Don't know/depends
- Unimportant
- Should not be included

* 164. The first aider should discuss the changes they have observed with those close to the person, with the person's permission.

- Essential
- Important
- Don't know/depends
- Unimportant
- Should not be included

* 165. If the person is an adolescent, the first aider should offer to stay with them while they contact and speak to a parent or other trusted adult.

- Essential
- Important
- Don't know/depends
- Unimportant
- Should not be included

166. Please provide any additional items or comments related to this section.

BEING SUPPORTIVE Continued

Please rate how important (from ‘essential’ to ‘should not be included’) you think it is that each statement be included in the guidelines.

Please keep our definitions in mind when responding to this section. You can access the definitions here.

Treating the person with dignity and respect

* 167. The first aider should always treat the person with respect.

- Essential
- Important
- Don't know/depends
- Unimportant
- Should not be included

* 168. The first aider should acknowledge to the person the courage it may have taken for them to talk to the first aider.

- Essential
- Important
- Don't know/depends
- Unimportant
- Should not be included

* 169. The first aider should support the person in making their own decisions about their mental health.

- Essential
- Important
- Don't know/depends
- Unimportant
- Should not be included

* 170. The first aider should avoid using patronising or trivialising statements when interacting with the person, e.g. "cheer up", "I'm sure it will pass" and "it could be worse".

- Essential
- Important
- Don't know/depends
- Unimportant
- Should not be included

* 171. The first aider should not dismiss or ridicule the person, even if what they are saying doesn't make sense to them.

- Essential
- Important
- Don't know/depends
- Unimportant
- Should not be included

* 172. If the first aider is communicating with the person while others are present, the first aider should not speak about the person as though they are not there.

- Essential
- Important
- Don't know/depends
- Unimportant
- Should not be included

* 173. Unless the person is a danger to themselves or others, the first aider should respect their privacy and right to confidentiality.

- Essential
- Important
- Don't know/depends
- Unimportant
- Should not be included

* 174. If the first aider thinks they need to share any of the information the person has told them, they should get the person's agreement before doing so.

- Essential
- Important
- Don't know/depends
- Unimportant
- Should not be included

* 175. The first aider should be tolerant of changes in the person's behaviour, unless their behaviour becomes dangerous or inappropriate.

- Essential
- Important
- Don't know/depends
- Unimportant
- Should not be included

* 176. The first aider should not express anger or frustration they may feel toward the person.

- Essential
- Important
- Don't know/depends
- Unimportant
- Should not be included

* 177. If the person is upset by something the first aider has said or done, the first aider should apologise and acknowledge the person's feelings.

- Essential
- Important
- Don't know/depends
- Unimportant
- Should not be included

* 178. The first aider should use statements that show their confidence in the person, e.g. "I know you'll do fine, you can handle it".

- Essential
- Important
- Don't know/depends
- Unimportant
- Should not be included

* 179. The first aider should not attempt to take over or make decisions for the person without their involvement.

- Essential
- Important
- Don't know/depends
- Unimportant
- Should not be included

180. Please provide any additional items or comments related to this section.

BEING SUPPORTIVE Continued

Please rate how important (from ‘essential’ to ‘should not be included’) you think it is that each statement be included in the guidelines.

Please keep our definitions in mind when responding to this section. You can access the definitions here.

Encouraging other supports

* 181. The first aider should ask the person if they have felt this way before, and if so, what they have done in the past that has been helpful.

- Essential
- Important
- Don't know/depends
- Unimportant
- Should not be included

* 182. The first aider should encourage the person to attend a support group.

- Essential
- Important
- Don't know/depends
- Unimportant
- Should not be included

* 183. The first aider should try to determine whether the person has a supportive social network and if they do, the first aider should encourage them to use these supports.

- Essential
- Important
- Don't know/depends
- Unimportant
- Should not be included

* 184. The first aider should encourage the person to continue with their usual social, recreational or work activities.

- Essential
- Important
- Don't know/depends
- Unimportant
- Should not be included

* 185. The first aider should encourage the person to try self-help strategies, e.g. relaxation methods, physical activity, good sleep habits.

- Essential
- Important
- Don't know/depends
- Unimportant
- Should not be included

* 186. The first aider should encourage the person to engage in a healthy lifestyle, e.g. regular exercise, healthy diet, not using substances.

- Essential
- Important
- Don't know/depends
- Unimportant
- Should not be included

* 187. The first aider should encourage the person to connect with a role model who can provide positive information about their own experiences.

- Essential
- Important
- Don't know/depends
- Unimportant
- Should not be included

* 188. The first aider should let the person know that there are programs that provide support for education and employment goals, if these are important to the person.

- Essential
- Important
- Don't know/depends
- Unimportant
- Should not be included

189. Please provide any additional items or comments related to this section.

SUBSTANCE USE

This section contains additional statements about what the first aider needs to know specifically in relation to substance use and psychosis.

Please rate how important (from ‘essential’ to ‘should not be included’) you think it is that each statement be included in the guidelines.

Please keep our definitions in mind when responding to this section. You can access the definitions here.

There is 1 part to this section.

Substance use

* 190. The first aider should discourage the person from using alcohol or other drugs, as these may worsen symptoms of psychosis.

- ◯ Essential
- ◯ Important
- ◯ Don't know/depends
- ◯ Unimportant
- ◯ Should not be included

* 191. The first aider should tell the person that alcohol and other drugs can make their symptoms worse.

- ◯ Essential
- ◯ Important
- ◯ Don't know/depends
- ◯ Unimportant
- ◯ Should not be included

192. Please provide any additional items or comments related to this section.

POSTNATAL PSYCHOSIS

This section contains <u>additional</u> statements about what the first aider needs to know <u>specifically</u> in relation to providing mental health first aid to someone who may be experiencing postnatal psychosis.

Please rate how important (from ‘essential’ to ‘should not be included’) you think it is that each statement be included in the guidelines.

Please keep our definitions in mind when responding to this section. You can access the definitions here.

There is 1 part to this section.

Postnatal psychosis

* 193. If the first aider thinks a mother may be experiencing postnatal psychosis, they should call a mental health crisis team immediately, as it can escalate rapidly and delays in treatment can lead to increased risk for the mother and her baby

- Essential
- Important
- Don't know/depends
- Unimportant
- Should not be included

* 194. If a mother has delusions that involve her baby, the first aider should call a mental health crisis team immediately.

- Essential
- Important
- Don't know/depends
- Unimportant
- Should not be included

* 195. If the first aider thinks a mother may be experiencing postnatal psychosis, they should ensure that someone is with her at all times until professional help is received.

- Essential
- Important
- Don't know/depends
- Unimportant
- Should not be included

* 196. The first aider should try to involve the mother's partner or family in minimising any risk to the mother or baby.

- Essential
- Important
- Don't know/depends
- Unimportant
- Should not be included

197. Please provide any additional items or comments related to this section.

ENCOURAGING PROFESSIONAL HELP (in a non-crisis situation)

This section contains statements about what the first aider needs to know about encouraging the person to seek professional help, when the person is NOT in crisis.

Please rate how important (from ‘essential’ to ‘should not be included’) you think it is that each statement be included in the guidelines.

Please keep our definitions in mind when responding to this section. You can access the definitions here.

There are 5 parts to this section:

- What the first aider should know about encouraging professional help
- Encouraging professional help - general
- Providing the person with information and resources about professional help
- Supporting the person to seek professional help
- If the person does seek professional help

What the first aider should know about encouraging professional help

* 198. The first aider should know what services are available locally.

- Essential
- Important
- Don't know/depends
- Unimportant
- Should not be included

* 199. The first aider should have some general knowledge about the types of treatment that can be helpful for psychosis.

- Essential
- Important
- Don't know/depends
- Unimportant
- Should not be included

* 200. The first aider should know about the local pathways to professional help, e.g. referral from a GP or family doctor in order to see a specialist.

- ◯ Essential
- ◯ Important
- ◯ Don't know/depends
- ◯ Unimportant
- ◯ Should not be included

201. Please provide any additional items or comments related to this section.

ENCOURAGING PROFESSIONAL HELP (in a non-crisis situation) Continued

Please rate how important (from ‘essential’ to ‘should not be included’) you think it is that each statement be included in the guidelines.

Please keep our definitions in mind when responding to this section. You can access the definitions here.

Encouraging professional help - general

* 202. The first aider should treat the person as responsible and capable of making decisions about whether and when to seek professional help.

- Essential
- Important
- Don't know/depends
- Unimportant
- Should not be included

* 203. The first aider should suggest to the person that they seek professional help.

- Essential
- Important
- Don't know/depends
- Unimportant
- Should not be included

* 204. The first aider should tell the person that what they are experiencing could improve with appropriate professional help.

- Essential
- Important
- Don't know/depends
- Unimportant
- Should not be included

* 205. When encouraging the person to seek professional help, the first aider should focus on particular symptoms that are concerning the person and how treatment may help.

- Essential
- Important
- Don't know/depends
- Unimportant
- Should not be included

* 206. The first aider should encourage the person to seek professional help by letting them know that if they were going through the same experience they would seek help.

- Essential
- Important
- Don't know/depends
- Unimportant
- Should not be included

* 207. The first aider should try to find out what type of professional help the person believes will help them.

- Essential
- Important
- Don't know/depends
- Unimportant
- Should not be included

* 208. The first aider should convey that seeking professional help as soon as possible is important to prevent symptoms from getting worse, without putting pressure on the person to seek help.

- Essential
- Important
- Don't know/depends
- Unimportant
- Should not be included

* 209. The first aider should encourage the person to seek appropriate professional help, as soon as possible, even if they are unsure whether the person is developing or experiencing psychosis.

- Essential
- Important
- Don't know/depends
- Unimportant
- Should not be included

* 210. The first aider should let the person know that their symptoms may be caused by a variety of medical conditions that require treatment.

- Essential
- Important
- Don't know/depends
- Unimportant
- Should not be included

* 211. Because symptoms of psychosis may stem from physical illnesses, the first aider should encourage the person to see their GP or family doctor for a check-up.

- Essential
- Important
- Don't know/depends
- Unimportant
- Should not be included

* 212. The first aider should convey a message of hope to the person by telling them that help is available and things can get better.

- Essential
- Important
- Don't know/depends
- Unimportant
- Should not be included

* 213. The first aider should continue encouraging the person to seek professional help, even if challenges arise in the process of the person obtaining care.

- Essential
- Important
- Don't know/depends
- Unimportant
- Should not be included

* 214. The first aider should not pressure the person to seek professional help, unless they are concerned for the person's safety.

- Essential
- Important
- Don't know/depends
- Unimportant
- Should not be included

* 215. The first aider should not be threatening or confrontational when encouraging the person to seek professional help.

- Essential
- Important
- Don't know/depends
- Unimportant
- Should not be included

216. Please provide any additional items or comments related to this section.

ENCOURAGING PROFESSIONAL HELP (in a non-crisis situation) Continued

Please rate how important (from 'essential' to 'should not be included') you think it is that each statement be included in the guidelines.

Please keep our definitions in mind when responding to this section. You can access the definitions here.

Providing the person with information and resources about professional help

* 217. The first aider should provide the person with a range of options for seeking professional help.

- ◯ Essential
- ◯ Important
- ◯ Don't know/depends
- ◯ Unimportant
- ◯ Should not be included

* 218. The first aider should provide the person with relevant resources (e.g. printed materials, websites, telephone numbers) and information about local services.

- ◯ Essential
- ◯ Important
- ◯ Don't know/depends
- ◯ Unimportant
- ◯ Should not be included

* 219. The first aider should let the person know that their GP or family doctor can be a good first point of contact when seeking professional help.

- ◯ Essential
- ◯ Important
- ◯ Don't know/depends
- ◯ Unimportant
- ◯ Should not be included

* 220. The first aider should ask the person whether they have a doctor they trust, and if they do, the first aider should encourage them to seek professional help from their doctor.

- Essential
- Important
- Don't know/depends
- Unimportant
- Should not be included

* 221. The first aider should let the person know that if they seek professional help from a GP or family doctor, they will receive an assessment and may be referred to specialist services.

- Essential
- Important
- Don't know/depends
- Unimportant
- Should not be included

* 222. If the person asks for advice or suggestions regarding treatment, the first aider should tell the person that they are not qualified to discuss treatment options.

- Essential
- Important
- Don't know/depends
- Unimportant
- Should not be included

223. Please provide any additional items or comments related to this section.

Please rate how important (from 'essential' to 'should not be included') you think it is that each statement be included in the guidelines.

Please keep our definitions in mind when responding to this section. You can access the definitions here.

Supporting the person to seek professional help

* 224. The first aider should reassure the person that it is okay to seek help and point out that seeking help is a sign of strength rather than a sign of weakness or failure.

- Essential
- Important
- Don't know/depends
- Unimportant
- Should not be included

* 225. The first aider should reassure the person that health professionals will be on their side.

- Essential
- Important
- Don't know/depends
- Unimportant
- Should not be included

* 226. The first aider should explain to the person that seeking professional help does not necessarily mean they will be hospitalised, as early treatment can take place in the community.

- Essential
- Important
- Don't know/depends
- Unimportant
- Should not be included

* 227. The first aider should explain to the person that a health professional must maintain confidentiality except in limited circumstances, e.g. if the person is at risk of harming themselves or others, or if directed to by a court.

- Essential
- Important
- Don't know/depends
- Unimportant
- Should not be included

* 228. The first aider should be aware of the influence that the person’s family may have, e.g. the family may encourage or discourage the person from obtaining the care that they need.

- Essential
- Important
- Don't know/depends
- Unimportant
- Should not be included

* 229. The first aider should offer to make an appointment for the person to see a health professional.

- Essential
- Important
- Don't know/depends
- Unimportant
- Should not be included

* 230. If the first aider makes an appointment on behalf of the person, they should request a longer one so the person will have adequate time to discuss their symptoms and concerns.

- Essential
- Important
- Don't know/depends
- Unimportant
- Should not be included

* 231. The first aider should ask the person if they would like the first aider, or another relative or friend, to accompany them to their appointment.

- Essential
- Important
- Don't know/depends
- Unimportant
- Should not be included

* 232. If appropriate to the relationship, the first aider should reassure the person that they will support them while they seek and receive professional help.

- Essential
- Important
- Don't know/depends
- Unimportant
- Should not be included

* 233. If the person is an adolescent, the first aider should offer to go with them when they seek professional help.

- Essential
- Important
- Don't know/depends
- Unimportant
- Should not be included

* 234. If the person is having difficulty getting advice or help, the first aider should encourage the person to contact a mental health advocacy or support agency.

- Essential
- Important
- Don't know/depends
- Unimportant
- Should not be included

ENCOURAGING PROFESSIONAL HELP (in a non-crisis situation) Continued

Please rate how important (from 'essential' to 'should not be included') you think it is that each statement be included in the guidelines.

Please keep our definitions in mind when responding to this section. You can access the definitions here.

Supporting the person to seek professional help - continued

* 235. If the first aider wants to talk to a professional about their concerns for the person, they should get the person's permission before doing so.

- Essential
- Important
- Don't know/depends
- Unimportant
- Should not be included

* 236. If the first aider provides any information to a health professional regarding the person, the first aider should tell the person what information they have shared.

- Essential
- Important
- Don't know/depends
- Unimportant
- Should not be included

* 237. The first aider should be aware that, while they are entitled to express their concerns about the person to the person's health professional and ask for their assistance, the health professional must maintain confidentiality and is therefore unlikely to provide any information.

- Essential
- Important
- Don't know/depends
- Unimportant
- Should not be included

* 238. If the person is having difficulty getting advice or help, the first aider should contact a mental health advocacy or support agency on behalf of the person.

- Essential
- Important
- Don't know/depends
- Unimportant
- Should not be included

* 239. If the person is an adolescent, the first aider should ensure that the adolescent gets an appointment to see a health professional.

- Essential
- Important
- Don't know/depends
- Unimportant
- Should not be included

240. Please provide any additional items or comments related to this section.

If the person does seek professional help

* 241. If the person agrees to seek professional help, the first aider should help them to write a list of questions or points that they want to discuss with their health professional.

- Essential
- Important
- Don't know/depends
- Unimportant
- Should not be included

* 242. If the person agrees to seek professional help, the first aider should encourage them to request a longer appointment so they will have adequate time to discuss their symptoms and concerns.

- Essential
- Important
- Don't know/depends
- Unimportant
- Should not be included

* 243. If the person agrees to see a GP or family doctor for a check-up, the first aider should contact the doctor to explain why they are concerned about the person.

- Essential
- Important
- Don't know/depends
- Unimportant
- Should not be included

* 244. If <u>the person</u> lacks confidence in the professional advice received, the first aider should encourage the person to seek a second opinion from another health professional.

- Essential
- Important
- Don't know/depends
- Unimportant
- Should not be included

* 245. If the <u>first aider</u> lacks confidence in the professional advice that the person has received, the first aider should encourage the person to seek a second opinion from another health professional.

- Essential
- Important
- Don't know/depends
- Unimportant
- Should not be included

246. Please provide any additional items or comments related to this section.

IF THE PERSON DOESN’T WANT PROFESSIONAL HELP (in a non-crisis situation)

This section contains statements about what the first aider needs to know about what to do when the person doesn’t want professional help, and the person is NOT in crises.

Please rate how important (from ‘essential’ to ‘should not be included’) you think it is that each statement be included in the guidelines.

Please keep our definitions in mind when responding to this section. You can access the definitions here.

There are 2 parts to this section:

- If the person doesn’t want professional help
- Engaging other people if the person doesn’t want professional help

If the person doesn’t want professional help

* 247. If the person does not want to seek professional help, the first aider should...

	Essential	Important	Don't know/depends	Unimportant	Should not be included
…remain patient, as people experiencing psychosis often need time to recognise that they are unwell.	○	○	○	○	○
…calmly express their concern to the person about their choice to not seek help and the potential implications.	○	○	○	○	○
…stress the potential benefits of getting help, such as relief from anxiety or frightening symptoms.	○	○	○	○	○
…be persistent in encouraging the person to seek help.	○	○	○	○	○
…be prepared to have several conversations with the person before they are willing to seek professional help.	○	○	○	○	○

	Essential	Important	Don't know/depends	Unimportant	Should not be included
…focus on trying to find something that the person agrees is a problem and then suggest that the person seek help for that, e.g. if the person says that they feel anxious around other people, the first aider should encourage them to seek help for anxiety.	○	○	○	○	○
…discuss their concerns with the person using examples of behaviour or problems they have noticed.	○	○	○	○	○
…explore the reasons for this, e.g. not realising they are unwell, worries about stigma, or not knowing where to get help.	○	○	○	○	○

* 248. The first aider should be aware that the person may not want professional help because they believe that others are trying to harm them.

- ○ Essential
- ○ Important
- ○ Don't know/depends
- ○ Unimportant
- ○ Should not be included

* 249. If the person does not want to seek professional help, the first aider should not try to convince the person that they are experiencing psychosis.

- ○ Essential
- ○ Important
- ○ Don't know/depends
- ○ Unimportant
- ○ Should not be included

* 250. The first aider should try to maintain a good relationship with the person, as they may want the first aider’s help in the future.

- Essential
- Important
- Don't know/depends
- Unimportant
- Should not be included

* 251. The first aider should be aware that the person has the right to refuse treatment, unless they meet the criteria for involuntary treatment.

- Essential
- Important
- Don't know/depends
- Unimportant
- Should not be included

* 252. The first aider should never threaten the person with involuntary treatment or hospitalisation.

- Essential
- Important
- Don't know/depends
- Unimportant
- Should not be included

253. Please provide any additional items or comments related to this section.

IF THE PERSON DOESN'T WANT PROFESSIONAL HELP (in a non-crisis situation) Continued

Please rate how important (from ‘essential’ to ‘should not be included’) you think it is that each statement be included in the guidelines.

Please keep our definitions in mind when responding to this section. You can access the definitions here.

Engaging other people if the person doesn’t want professional help

* 254. The first aider should seek advice from others who have experience encouraging a person to seek professional help for psychotic symptoms.

- Essential
- Important
- Don't know/depends
- Unimportant
- Should not be included

* 255. The first aider should talk to the person’s family or close friends, as they may be able to facilitate the next step towards obtaining professional help for the person

- Essential
- Important
- Don't know/depends
- Unimportant
- Should not be included

* 256. The first aider should encourage them to talk to someone they trust about what they are experiencing.

- Essential
- Important
- Don't know/depends
- Unimportant
- Should not be included

* 257. The first aider should consult with experts for advice on how best to support the person to seek professional help.

- Essential
- Important
- Don't know/depends
- Unimportant
- Should not be included

* 258. If the first aider shares information about the person with a health professional, they should request that this is kept confidential and used as sensitively as possible in order to protect their relationship with the person.

- Essential
- Important
- Don't know/depends
- Unimportant
- Should not be included

* 259. If the first aider does discuss their concerns about the person with a health professional, they should clearly describe their observations (e.g. exactly what the person has been doing and saying, where and when) so that they have all the necessary information.

- Essential
- Important
- Don't know/depends
- Unimportant
- Should not be included

* 260. If the person does not want to seek professional help, the first aider should let the person know about other options for help in their community, e.g. a home visit or community services.

- Essential
- Important
- Don't know/depends
- Unimportant
- Should not be included

* 261. If the person does not recognise that they are unwell, the first aider should be aware that they might actively resist the first aider’s attempts to encourage them to seek help.

- Essential
- Important
- Don't know/depends
- Unimportant
- Should not be included

262. Please provide any additional items or comments related to this section.

HALLUCINATIONS AND DELUSIONS (in a non-crisis situation)

This section contains statements about what the first aider needs to know about hallucinations and delusions, and how to respond to them. It specifically relates to situations in which a person is or has been experiencing hallucinations or delusion <u>but is NOT in crisis.</u>

Please rate how important (from ‘essential’ to ‘should not be included’) you think it is that each statement be included in the guidelines.

Please keep our definitions in mind when responding to this section. You can access the definitions here.

There are 3 parts to this section:

- Finding out about what the person is experiencing
- Responding to hallucinations and delusions
- If the person is paranoid

Finding out about what the person is experiencing

* 263. The first aider should know that delusions or hallucinations are very real to the person.

- ◯ Essential
- ◯ Important
- ◯ Don't know/depends
- ◯ Unimportant
- ◯ Should not be included

* 264. If the person wants to talk about their hallucinations or delusions, the first aider should listen in order to demonstrate empathy and develop an understanding of what they are experiencing.

- ◯ Essential
- ◯ Important
- ◯ Don't know/depends
- ◯ Unimportant
- ◯ Should not be included

* 265. The first aider should ask the person if they want to talk about what they are seeing or hearing.

- Essential
- Important
- Don't know/depends
- Unimportant
- Should not be included

* 266. The first aider should encourage discussion of the person's feelings rather than the content of their hallucinations or delusions.

- Essential
- Important
- Don't know/depends
- Unimportant
- Should not be included

* 267. If the person is finding it challenging describing their hallucinations or delusions, the first aider should use other creative ways to help the person describe their experience, e.g. painting or acting.

- Essential
- Important
- Don't know/depends
- Unimportant
- Should not be included

* 268. The first aider should encourage the person to consider the evidence for their delusion by asking questions in a non-judgmental and non-challenging way.

- Essential
- Important
- Don't know/depends
- Unimportant
- Should not be included

* 269. The first aider should ask questions about the content of the person’s delusions, particularly any elements that indicate the potential for harming themselves or others.

- Essential
- Important
- Don't know/depends
- Unimportant
- Should not be included

* 270. Until the first aider knows the content and context of the person’s delusions, it is important to keep themselves safe from potentially aggressive reactions.

- Essential
- Important
- Don't know/depends
- Unimportant
- Should not be included

271. Please provide any additional items or comments related to this section.

HALLUCINATIONS AND DELUSIONS (in a non-crisis situation) Continued

Please rate how important (from ‘essential’ to ‘should not be included’) you think it is that each statement be included in the guidelines.

Please keep our definitions in mind when responding to this section. You can access the definitions here.

Responding to hallucinations and delusions

* 272. The first aider should know that if the person is hearing voices, they may react through behaviours – such as talking to oneself, whispering to oneself – in response to the voices they are hearing.

- Essential
- Important
- Don't know/depends
- Unimportant
- Should not be included

* 273. The first aider should gently and matter-of-factly tell the person that the content of their delusions is not common or real and that they do not share them.

- Essential
- Important
- Don't know/depends
- Unimportant
- Should not be included

* 274. The first aider should acknowledge to the person that what they are experiencing is real to them, without confirming or denying their hallucinations or delusions, e.g. by stating "I accept that you hear voices or see things in that way, but it's not like that for me."

- Essential
- Important
- Don't know/depends
- Unimportant
- Should not be included

* 275. The first aider should try to empathise with how the person feels about their beliefs and experiences, without stating any judgments about the content of those beliefs and experiences.

- Essential
- Important
- Don't know/depends
- Unimportant
- Should not be included

* 276. The first aider should validate any part of the hallucination or delusion that is real, e.g. "Yes there was a person standing over there, but I did not hear him talking about you".

- Essential
- Important
- Don't know/depends
- Unimportant
- Should not be included

* 277. The first aider should not:

	Essential	Important	Don't know/depends	Unimportant	Should not be included
...pretend to agree with the person's hallucinations or delusions.					
...try to reason with the person about their hallucinations or delusions.					
...dismiss, minimise or argue with the person about their delusions or hallucinations					
...act alarmed or embarrassed by the person's hallucinations or delusions.					
...laugh at or make fun of the person's delusions or hallucinations.					

* 278. The first aider should encourage the person to tell the voices to go away.

- Essential
- Important
- Don't know/depends
- Unimportant
- Should not be included

* 279. If the first aider feels uncomfortable, upset or embarrassed by the person's hallucinations or delusions, they should tell the person they do not want to talk about these.

- Essential
- Important
- Don't know/depends
- Unimportant
- Should not be included

* 280. If the first aider feels uncomfortable, upset or embarrassed by the person's hallucinations or delusions, they should tell the person this.

- Essential
- Important
- Don't know/depends
- Unimportant
- Should not be included

HALLUCINATIONS AND DELUSIONS (in a non-crisis situation) Continued

Please rate how important (from ‘essential’ to ‘should not be included’) you think it is that each statement be included in the guidelines.

Please keep our definitions in mind when responding to this section. You can access the definitions here.

Responding to hallucinations and delusions - continued

* 281. The first aider should encourage the person to read about hallucinations or delusions from a reputable source.

- Essential
- Important
- Don't know/depends
- Unimportant
- Should not be included

* 282. The first aider should ask the person if they are afraid or confused.

- Essential
- Important
- Don't know/depends
- Unimportant
- Should not be included

* 283. The first aider should let the person know that many people experience hearing voices.

- Essential
- Important
- Don't know/depends
- Unimportant
- Should not be included

* 284. The first aider should try to distract the person from their hallucinations or delusions, e.g. by involving the person in something interesting.

- Essential
- Important
- Don't know/depends
- Unimportant
- Should not be included

* 285. If appropriate, the first aider should try to use humour to calm the person.

- Essential
- Important
- Don't know/depends
- Unimportant
- Should not be included

* 286. If appropriate, the first aider should use gentle physical touch to calm the person.

- Essential
- Important
- Don't know/depends
- Unimportant
- Should not be included

* 287. The first aider should tell the person it is possible for hallucinations or delusions to go away.

- Essential
- Important
- Don't know/depends
- Unimportant
- Should not be included

* 288. The first aider should know that it is not helpful to encourage the person to try to stop hallucinations.

- Essential
- Important
- Don't know/depends
- Unimportant
- Should not be included

* 289. If it is appropriate to their relationship, the first aider should let the person know they love and support them, as this can help the person to feel safe.

- Essential
- Important
- Don't know/depends
- Unimportant
- Should not be included

* 290. If the first aider observes other people making jokes about or criticising the person, they should tell them to stop.

- Essential
- Important
- Don't know/depends
- Unimportant
- Should not be included

* 291. The first aider should avoid using the terms "hallucinations" or "delusions" when speaking to the person, as these terms may imply dismissal of the person’s ideas and experiences.

- Essential
- Important
- Don't know/depends
- Unimportant
- Should not be included

* 292. The first aider should ask the person if there is anything they have found that reduces their hallucinations or delusions, and if there is, encourage the person to use these strategies.

- Essential
- Important
- Don't know/depends
- Unimportant
- Should not be included

* 293. If there are aspects of the person's environment that seem to increase their hallucinations or delusions, the first aider should limit or remove these where possible.

- Essential
- Important
- Don't know/depends
- Unimportant
- Should not be included

294. Please provide any additional items or comments related to this section.

HALLUCINATIONS AND DELUSIONS (in a non-crisis situation) Continued

Please rate how important (from ‘essential’ to ‘should not be included’) you think it is that each statement be included in the guidelines.

Please keep our definitions in mind when responding to this section. You can access the definitions here.

If the person is paranoid

* 295. The first aider should be aware that the person's experience of hallucinations or delusions may cause them not to trust people, even those close to them.

- Essential
- Important
- Don't know/depends
- Unimportant
- Should not be included

* 296. If the person exhibits paranoia, the first aider should not encourage or inflame this, e.g. by whispering to or about them.

- Essential
- Important
- Don't know/depends
- Unimportant
- Should not be included

* 297. If the person is experiencing paranoia, the first aider should:

	Essential	Important	Don't know/depends	Unimportant	Should not be included
...ask the person about their fears.	○	○	○	○	○
...give the person simple directions, if needed, e.g. "sit down, and let's talk about it".	○	○	○	○	○
...tell the person that no harm will come to them.	○	○	○	○	○
...stay with the person, but at a distance that is comfortable for both the person and the first aider.	○	○	○	○	○
...encourage and support the person to move away from whatever is causing their fear, if it is safe to do so.	○	○	○	○	○
...tell the person that they themselves are not afraid of what is causing the person fear.	○	○	○	○	○
...tell the person what they are going to do before doing it, e.g. that they are going to get out their phone.	○	○	○	○	○

298. Please provide any additional items or comments related to this section.

ASSESSING WHETHER THE PERSON IS IN CRISIS

This section contains statements about what the first aider needs to know about assessing whether the person IS in crisis.

Please rate how important (from ‘essential’ to ‘should not be included’) you think it is that each statement be included in the guidelines.

Please keep our definitions in mind when responding to this section. You can access the definitions here.

There is 1 part to this section.

Assessing whether the person is in crisis.

* 299. The first aider should assess for risk of harm to the person or others.

- Essential
- Important
- Don't know/depends
- Unimportant
- Should not be included

* 300. If the first aider does not think the person is in immediate risk of harm, but is still concerned about their welfare, they should ask the person if there is someone close to them who may be able to support them to stay safe.

- Essential
- Important
- Don't know/depends
- Unimportant
- Should not be included

* 301. If the first aider thinks the person is at risk of suicide, the first aider should follow the Mental Health First Aid Guidelines for Suicidal Thoughts and Behaviours.

- ◯ Essential
- ◯ Important
- ◯ Don't know/depends
- ◯ Unimportant
- ◯ Should not be included

302. Please provide any additional items or comments related to this section.

[ ]

WHEN THE PERSON IS IN CRISIS (is in a severe psychotic state OR behaving aggressively)

This section contains statements about what the first aider needs to know about what to do when the person is in crisis (that is, they are in a severe psychotic state or are behaving aggressively, but the first aider does not think the person is at risk of suicide).

Please rate how important (from 'essential' to 'should not be included') you think it is that each statement be included in the guidelines.

Please keep our definitions in mind when responding to this section. You can access the definitions here.

There are 5 parts to this section:

- Safety considerations when the person is in a severe psychotic state or behaving aggressively
- Communicating with the person when they are in a severe psychotic state or behaving aggressively
- De-escalation when the person is in a severe psychotic state or behaving aggressively
- Seeking help for the person when they are in a severe psychotic state or behaving aggressively
- Calling emergency services for help when the person is in a severe psychotic state or behaving aggressively

Safety considerations when the person is in a severe psychotic state or behaving aggressively

* 303. The first aider's primary goal should be to keep themselves and others safe.

	Essential	Important	Don't know/depends	Unimportant	Should not be included
Severe psychotic state	○	○	○	○	○
Behaving aggressively	○	○	○	○	○

* 304. The first aider should take any threats or warnings seriously, particularly if the person believes they are being persecuted.

	Essential	Important	Don't know/depends	Unimportant	Should not be included
Severe psychotic state	○	○	○	○	○
Behaving aggressively	○	○	○	○	○

* 305. If the first aider is frightened, they should seek outside help immediately, as they should never put themselves at risk.

	Essential	Important	Don't know/depends	Unimportant	Should not be included
Severe psychotic state	○	○	○	○	○
Behaving aggressively	○	○	○	○	○

* 306. The first aider should be aware that the person might act upon a hallucination or delusion.

	Essential	Important	Don't know/depends	Unimportant	Should not be included
Severe psychotic state	◯	◯	◯	◯	◯
Behaving aggressively	◯	◯	◯	◯	◯

* 307. The first aider should approach the person with caution.

	Essential	Important	Don't know/depends	Unimportant	Should not be included
Severe psychotic state	◯	◯	◯	◯	◯
Behaving aggressively	◯	◯	◯	◯	◯

* 308. If the person has a weapon or something that could be used as weapon, the first aider should not approach the person and should call emergency services immediately.

	Essential	Important	Don't know/depends	Unimportant	Should not be included
Severe psychotic state	◯	◯	◯	◯	◯
Behaving aggressivey	◯	◯	◯	◯	◯

* 309. The first aider should try to gather information about whether the person feels safe, e.g. by stating "You seem worried; is there anything I can do to help?" or "Do you feel safe? Or is there something you are afraid of?"

	Essential	Important	Don't know/depends	Unimportant	Should not be included
Severe psychotic state	◯	◯	◯	◯	◯
Behaving aggressively	◯	◯	◯	◯	◯

* 310. The first aider should try to protect the person, themselves, and others around them from harm.

	Essential	Important	Don't know/depends	Unimportant	Should not be included
Severe psychotic state	◯	◯	◯	◯	◯
Behaving aggressively	◯	◯	◯	◯	◯

* 311. If the person threatens to harm themselves or others, the first aider should try to secure the person in a room while they seek help.

	Essential	Important	Don't know/depends	Unimportant	Should not be included
Severe psychotic state	◯	◯	◯	◯	◯
Behaving aggressively	◯	◯	◯	◯	◯

* 312. The first aider should stay at a safe distance from the person while being able to maintain interaction.

	Essential	Important	Don't know/depends	Unimportant	Should not be included
Severe psychotic state	○	○	○	○	○
Behaving aggressively	○	○	○	○	○

* 313. The first aider should ensure they have clear access to an exit.

	Essential	Important	Don't know/depends	Unimportant	Should not be included
Severe psychotic state	○	○	○	○	○
Behaving aggressively	○	○	○	○	○

* 314. If the person is upset and suddenly stands up, the first aider should also stand up so they can leave quickly if the situation gets dangerous.

	Essential	Important	Don't know/depends	Unimportant	Should not be included
Severe psychotic state	○	○	○	○	○
Behaving aggressively	○	○	○	○	○

* 315. If safe to do so, the first aider should try to limit access to means that the person could use to harm themselves or others.

	Essential	Important	Don't know/depends	Unimportant	Should not be included
Severe psychotic state	○	○	○	○	○
Behaving aggressively	○	○	○	○	○

* 316. If safe to do so, the first aider should remove any weapons or objects that could be used as weapons from the person's immediate environment.

	Essential	Important	Don't know/depends	Unimportant	Should not be included
Severe psychotic state	○	○	○	○	○
Behaving aggressively	○	○	○	○	○

* 317. The first aider should comply with requests, unless they are unreasonable or unsafe, as this gives the person the opportunity to feel somewhat in control.

	Essential	Important	Don't know/depends	Unimportant	Should not be included
Severe psychotic state	○	○	○	○	○
Behaving aggressively	○	○	○	○	○

* 318. If the first aider is alone with the person, they should call another person to accompany the first aider until professional help arrives.

	Essential	Important	Don't know/depends	Unimportant	Should not be included
Severe psychotic state	○	○	○	○	○
Behaving aggressively	○	○	○	○	○

* 319. If the first aider is alone with the person and cannot stay, they should call someone to stay with the person until professional help arrives.

	Essential	Important	Don't know/depends	Unimportant	Should not be included
Severe psychotic state	○	○	○	○	○
Behaving aggressively	○	○	○	○	○

* 320. If the first aider does not feel it is safe for them to take the person to a hospital, the first aider should call emergency services.

	Essential	Important	Don't know/depends	Unimportant	Should not be included
Severe psychotic state	○	○	○	○	○
Behaving aggressively	○	○	○	○	○

321. Please provide any additional items or comments related to this section.

WHEN THE PERSON IS IN CRISIS (is in a severe psychotic state OR behaving aggressively) Continued

Please rate how important (from 'essential' to 'should not be included') you think it is that each statement be included in the guidelines.

Please keep our definitions in mind when responding to this section. You can access the definitions here.

Communicating with the person when they are in a severe psychotic state or behaving aggressively

* 322. The first aider should speak calmly.

	Essential	Important	Don't know/depends	Unimportant	Should not be included
Severe psychotic state	○	○	○	○	○
Behaving aggressively	○	○	○	○	○

* 323. The first aider should communicate in short, simple sentences.

	Essential	Important	Don't know/depends	Unimportant	Should not be included
Severe psychotic state	○	○	○	○	○
Behaving aggressively	○	○	○	○	○

* 324. The first aider should not shout at the person.

	Essential	Important	Don't know/depends	Unimportant	Should not be included
Severe psychotic state	○	○	○	○	○
Behaving aggressively	○	○	○	○	○

* 325. The first aider should not raise their voice.

	Essential	Important	Don't know/depends	Unimportant	Should not be included
Severe psychotic state	○	○	○	○	○
Behaving aggressively	○	○	○	○	○

* 326. The first aider should repeat questions or statements when necessary, using the same words each time rather than re-phrasing in the hope it will make things clearer for the person.

	Essential	Important	Don't know/depends	Unimportant	Should not be included
Severe psychotic state	◯	◯	◯	◯	◯
Behaving aggressively	◯	◯	◯	◯	◯

* 327. The first aider should repeat questions or statements when necessary, using different wording in the hope it will make things clearer for the person.

	Essential	Important	Don't know/depends	Unimportant	Should not be included
Severe psychotic states	◯	◯	◯	◯	◯
Behaving aggressively	◯	◯	◯	◯	◯

* 328. The first aider should try to minimise the level of emotion they show.

	Essential	Important	Don't know/depends	Unimportant	Should not be included
Severe psychotic state	◯	◯	◯	◯	◯
Behaving aggressively	◯	◯	◯	◯	◯

* 329. The first aider should avoid direct, continuous eye contact with the person.

	Essential	Important	Don't know/depends	Unimportant	Should not be included
Severe psychotic state	◯	◯	◯	◯	◯
Behaving aggressively	◯	◯	◯	◯	◯

* 330. The first aider should position themselves at the level of the person if it is safe to do so.

	Essential	Important	Don't know/depends	Unimportant	Should not be included
Severe psychotic state	◯	◯	◯	◯	◯
Behaving aggressively	◯	◯	◯	◯	◯

* 331. The first aider should sit down and encourage the person to sit down too.

	Essential	Important	Don't know/depends	Unimportant	Should not be included
Severe psychotic state	◯	◯	◯	◯	◯
Behaving aggressively	◯	◯	◯	◯	◯

* 332. The first aider should attempt to sit next to the person, if it is safe to do so.

	Essential	Important	Don't know/depends	Unimportant	Should not be included
Severe psychotic state	○	○	○	○	○
Behaving aggressively	○	○	○	○	○

* 333. The first aider should avoid touching the person.

	Essential	Important	Don't know/depends	Unimportant	Should not be included
Severe psychotic state	○	○	○	○	○
Behaving aggressively	○	○	○	○	○

* 334. If it is necessary to move close to or make physical contact with the person, the first aider should first ask the person for permission, e.g. "do you mind if I sit next to you?" or "I can see your arm is hurt, is it ok if I use the first-aid kit to bandage it?".

	Essential	Important	Don't know/depends	Unimportant	Should not be included
Severe psychotic state	○	○	○	○	○
Behaving aggressively	○	○	○	○	○

* 335. The first aider should try not take anything the person says personally.

	Essential	Important	Don't know/depends	Unimportant	Should not be included
Severe psychotic state	○	○	○	○	○
Behaving aggressively	○	○	○	○	○

336. Please provide any additional items or comments related to this section.

WHEN THE PERSON IS IN CRISIS (is in a severe psychotic state OR behaving aggressively) Continued

Please rate how important (from ‘essential’ to ‘should not be included’) you think it is that each statement be included in the guidelines.

Please keep our definitions in mind when responding to this section. You can access the definitions here.

De-escalation when the person is in a severe psychotic state or behaving aggressively

* 337. The first aider should try to de-escalate the situation.

	Essential	Important	Don't know/depends	Unimportant	Should not be included
Severe psychotic state	◯	◯	◯	◯	◯
Behaving aggressively	◯	◯	◯	◯	◯

* 338. The first aider should let the person know they are there to help.

	Essential	Important	Don't know/depends	Unimportant	Should not be included
Severe psychotic state	◯	◯	◯	◯	◯
Behaving aggressively	◯	◯	◯	◯	◯

* 339. The first aider should attempt to find out from the person what would help them feel safe and in control.

	Essential	Important	Don't know/depends	Unimportant	Should not be included
Severe psychotic state	◯	◯	◯	◯	◯
Behaving aggressively	◯	◯	◯	◯	◯

* 340. The first aider should try to remain calm.

	Essential	Important	Don't know/depends	Unimportant	Should not be included
Severe psychotic state	◯	◯	◯	◯	◯
Behaving aggressively	◯	◯	◯	◯	◯

* 341. The first aider should try not to show fear or anxiety.

	Essential	Important	Don't know/depends	Unimportant	Should not be included
Severe psychotic state	○	○	○	○	○
Behaving aggressively	○	○	○	○	○

* 342. The first aider should stay calm and avoid nervous behaviour, e.g. shuffling their feet, fidgeting, making abrupt movements, talking fast.

	Essential	Important	Don't know/depends	Unimportant	Should not be included
Severe psychotic state	○	○	○	○	○
Behaving aggressively	○	○	○	○	○

* 343. The first aider should allow the person to express their feelings.

	Essential	Important	Don't know/depends	Unimportant	Should not be included
Severe psychotic state	○	○	○	○	○
Behaving aggressively	○	○	○	○	○

* 344. The first aider should listen to the person more than talking to them.

	Essential	Important	Don't know/depends	Unimportant	Should not be included
Severe psychotic state	○	○	○	○	○
Behaving aggressively	○	○	○	○	○

* 345. The first aider should empathise with how the person is feeling.

	Essential	Important	Don't know/depends	Unimportant	Should not be included
Severe psychotic state	○	○	○	○	○
Behaving aggressively	○	○	○	○	○

* 346. The first aider should ask the person what they can do to help.

	Essential	Important	Don't know/depends	Unimportant	Should not be included
Severe psychotic state	○	○	○	○	○
Behaving aggressively	○	○	○	○	○

* 347. The first aider should try to find out if the person has anyone they trust (e.g. close friends or family) and, if so, the first aider should try to enlist their help.

	Essential	Important	Don't know/depends	Unimportant	Should not be included
Severe psychotic state	○	○	○	○	○
Behaving aggressively	○	○	○	○	○

* 348. The first aider should not do anything to further agitate the person.

	Essential	Important	Don't know/depends	Unimportant	Should not be included
Severe psychotic state	○	○	○	○	○
Behaving aggressively	○	○	○	○	○

* 349. The first aider should not try to restrict or restrain the person’s movement.

	Essential	Important	Don't know/depends	Unimportant	Should not be included
Severe psychotic state	○	○	○	○	○
Behaving aggressively	○	○	○	○	○

WHEN THE PERSON IS IN CRISIS (is in a severe psychotic state OR behaving aggressively) Continued

Please rate how important (from 'essential' to 'should not be included') you think it is that each statement be included in the guidelines.

Please keep our definitions in mind when responding to this section. You can access the definitions here.

De-escalation when the person is in a severe psychotic state or behaving aggressively - continued

* 350. The first aider should not argue with any other people present about the best course of action.

	Essential	Important	Don't know/depends	Unimportant	Should not be included
Severe psychotic state	◯	◯	◯	◯	◯
Behaving aggressively	◯	◯	◯	◯	◯

* 351. If there is more than one person present, the first aider should encourage only one person to speak at a time.

	Essential	Important	Don't know/depends	Unimportant	Should not be included
Severe psychotic state	◯	◯	◯	◯	◯
Behaving aggressively	◯	◯	◯	◯	◯

* 352. If there is more than one person present, the first aider should try to create a space around the person so that they don't feel crowded.

	Essential	Important	Don't know/depends	Unimportant	Should not be included
Severe psychotic state	◯	◯	◯	◯	◯
Behaving aggressively	◯	◯	◯	◯	◯

* 353. If there are people present who do not have a role in helping with the crisis, the first aider should ask them to leave.

	Essential	Important	Don't know/depends	Unimportant	Should not be included
Severe psychotic state	◯	◯	◯	◯	◯
Behaving aggressively	◯	◯	◯	◯	◯

* 354. If the person is consuming alcohol or other drugs, the first aider should discourage them from taking any more.

	Essential	Important	Don't know/depends	Unimportant	Should not be included
Severe psychotic state	◯	◯	◯	◯	◯
Behaving aggressively	◯	◯	◯	◯	◯

* 355. If the person is consuming alcohol or other drugs, the first aider should try to limit their access to these.

	Essential	Important	Don't know/depends	Unimportant	Should not be included
Severe psychotic state	◯	◯	◯	◯	◯
Behaving aggressively	◯	◯	◯	◯	◯

* 356. If feasible, the first aider should offer the person food or drink.

	Essential	Important	Don't know/depends	Unimportant	Should not be included
Severe psychotic state	◯	◯	◯	◯	◯
Behaving aggressively	◯	◯	◯	◯	◯

* 357. If the first aider is not able to de-escalate the situation, they should call for professional assistance, e.g. a mental health crisis service or emergency services.

	Essential	Important	Don't know/depends	Unimportant	Should not be included
Severe psychotic state	◯	◯	◯	◯	◯
Behaving aggressively	◯	◯	◯	◯	◯

358. Please provide any additional items or comments related to this section.

Seeking help for the person when they are in a severe psychotic state or behaving aggressively

* 359. If the person is receiving professional help for psychosis, the first aider should contact the person's health professional immediately.

	Essential	Important	Don't know/depends	Unimportant	Should not be included
Severe psychotic state	◯	◯	◯	◯	◯
Behaving aggressively	◯	◯	◯	◯	◯

* 360. If the first aider needs to call emergency services, they should explain this to the person before doing so.

	Essential	Important	Don't know/depends	Unimportant	Should not be included
Severe psychotic state	◯	◯	◯	◯	◯
Behaving aggressively	◯	◯	◯	◯	◯

* 361. If the first aider's concerns about the person are dismissed by the services they contact they should persevere in trying to seek support for the person, e.g. call another service.

	Essential	Important	Don't know/depends	Unimportant	Should not be included
Severe psychotic state	◯	◯	◯	◯	◯
Behaving aggressively	◯	◯	◯	◯	◯

* 362. If the person threatens to harm themselves or others, the first aider should not attempt to drive the person to the hospital without the support of others.

	Essential	Important	Don't know/depends	Unimportant	Should not be included
Severe psychotic state	◯	◯	◯	◯	◯
Behaving aggressively	◯	◯	◯	◯	◯

363. Please provide any additional items or comments related to this section.

WHEN THE PERSON IS IN CRISIS (is in a severe psychotic state OR behaving aggresively) Continued

Please rate how important (from ‘essential’ to ‘should not be included’) you think it is that each statement be included in the guidelines.

Please keep our definitions in mind when responding to this section. You can access the definitions here.

Calling for help when the person is in a severe psychotic state or behaving aggressively

* 364. If the first aider contacts a mental health service, they should not label the person as 'psychotic', but rather outline any symptoms and immediate concerns.

	Essential	Important	Don't know/depends	Unimportant	Should not be included
Severe psychotic state	○	○	○	○	○
Behaving aggressively	○	○	○	○	○

* 365. If the first aider suspects the person may be a threat to themselves or to others, the first aider should contact emergency services immediately.

	Essential	Important	Don't know/depends	Unimportant	Should not be included
Severe psychotic state	○	○	○	○	○
Behaving aggressively	○	○	○	○	○

* 366. The first aider should not use calling emergency services as a threat.

	Essential	Important	Don't know/depends	Unimportant	Should not be included
Severe psychotic state	○	○	○	○	○
Behaving aggressively	○	○	○	○	○

* 367. If the first aider calls emergency services they should explain that they are concerned the person may be experiencing psychosis.

	Essential	Important	Don't know/depends	Unimportant	Should not be included
Severe psychotic state	○	○	○	○	○
Behaving aggressively	○	○	○	○	○

* 368. If the first aider calls emergency services and the person has previously been diagnosed with a psychotic illness, the first aider should explain this.

	Essential	Important	Don't know/depends	Unimportant	Should not be included
Severe psychotic state	◯	◯	◯	◯	◯
Behaving aggressively	◯	◯	◯	◯	◯

* 369. If the first aider calls emergency services they should describe specific, concise observations about the person's behaviour and symptoms.

	Essential	Important	Don't know/depends	Unimportant	Should not be included
Severe psychotic state	◯	◯	◯	◯	◯
Behaving aggressively	◯	◯	◯	◯	◯

* 370. If the first aider calls emergency services they should let them know if the person is armed or if there are accessible weapons nearby.

	Essential	Important	Don't know/depends	Unimportant	Should not be included
Severe psychotic state	◯	◯	◯	◯	◯
Behaving aggressively	◯	◯	◯	◯	◯

* 371. If the first aider calls emergency services and the police respond, the first aider should be prepared that the person may be restrained or face charges.

	Essential	Important	Don't know/depends	Unimportant	Should not be included
Severe psychotic state	◯	◯	◯	◯	◯
Behaving aggressively	◯	◯	◯	◯	◯

* 372. If the first aider thinks the person is at risk of suicide or harming themselves, they should tell emergency services this.

	Essential	Important	Don't know/depends	Unimportant	Should not be included
Severe psychotic state	◯	◯	◯	◯	◯
Behaving aggressively	◯	◯	◯	◯	◯

* 373. If emergency services respond, the first aider should try to meet them on arrival so they can explain the situation before they approach the person.

	Essential	Important	Don't know/depends	Unimportant	Should not be included
Severe psychotic state	○	○	○	○	○
Behaving aggressively	○	○	○	○	○

* 374. If other people arrive, the first aider should explain to the person who they are, that they are there to help, and how they are going to help.

	Essential	Important	Don't know/depends	Unimportant	Should not be included
Severe psychotic state	○	○	○	○	○
Behaving aggressively	○	○	○	○	○

375. Please provide any additional items or comments related to this section.

SEVERE PSYCHOTIC STATES (crisis situation)

This section contains statements that pertain only to severe psychotic states

Please rate how important (from ‘essential’ to ‘should not be included’) you think it is that each statement be included in the guidelines.

Please keep our definitions in mind when responding to this section. You can access the definitions here.

There are 6 parts to this section:

- Communicating with the person when they are in a severe psychotic state
- De-escalation when the person is in a severe psychotic state
- Encouraging professional help when the person is in a severe psychotic state
- If the person doesn’t want professional help but is in a severe psychotic state
- Contacting emergency services when the person is in a severe psychotic state
- If the person is in a severe psychotic state and needs to go to hospital

Communicating with the person when they are in a severe psychotic state.

* 376. The first aider should know that they cannot reason with someone who is in an acute state of psychosis.

- ○ Essential
- ○ Important
- ○ Don't know/depends
- ○ Unimportant
- ○ Should not be included

377. Please provide any additional items or comments related to this section.

De-escalation when the person is in a severe psychotic state

* 378. The first aider should ask the person whether they would like them to decrease distractions and stimulation, e.g. turn off TV, reduce room lights.

- Essential
- Important
- Don't know/depends
- Unimportant
- Should not be included

* 379. If the person has unrealistic fears for their safety, the first aider should reassure them that they are safe.

- Essential
- Important
- Don't know/depends
- Unimportant
- Should not be included

* 380. If the person has an advance directive/relapse prevention plan, the first aider should follow this.

- Essential
- Important
- Don't know/depends
- Unimportant
- Should not be included

381. Please provide any additional items or comments related to this section.

Encouraging professional help when the person is in a severe psychotic state

* 382. The first aider should try to make sure the person is evaluated by a health professional immediately.

- Essential
- Important
- Don't know/depends
- Unimportant
- Should not be included

* 383. The first aider should provide the person with options regarding seeking professional help, as this may give the person a sense of control, e.g. "do you want to go to the hospital with me or would you prefer John to take you".

- Essential
- Important
- Don't know/depends
- Unimportant
- Should not be included

* 384. The first aider should explain to the person why they believe that a medical or mental health assessment is necessary.

- Essential
- Important
- Don't know/depends
- Unimportant
- Should not be included

385. Please provide any additional items or comments related to this section.

SEVERE PSYCHOTIC STATES (crisis situation) Continued

Please rate how important (from ‘essential’ to ‘should not be included’) you think it is that each statement be included in the guidelines.

Please keep our definitions in mind when responding to this section. You can access the definitions here.

If the person doesn’t want professional help but is in a severe psychotic state

* 386. The first aider should know that if the person is in a severe psychotic state and refuses to seek professional help, the person's nearest relative can request that a mental health assessment is carried out.

- Essential
- Important
- Don't know/depends
- Unimportant
- Should not be included

* 387. If the person is in a severe psychotic state and denies that they are unwell, the first aider should contact emergency services.

- Essential
- Important
- Don't know/depends
- Unimportant
- Should not be included

* 388. If the person is in a severe psychotic state and denies that they are unwell, the first aider should contact the emergency services and ask for an assessment under the relevant mental health legislation.

- Essential
- Important
- Don't know/depends
- Unimportant
- Should not be included

389. Please provide any additional items or comments related to this section.

Contacting emergency services when the person is in a severe psychotic state

* 390. If the person is in a severe psychotic state and the first aider calls emergency services:

	Essential	Important	Don't know/depends	Unimportant	Should not be included
...they should explain that the person is in urgent need of medical help.	○	○	○	○	○
...they should be aware that the person won't necessarily be admitted to hospital.	○	○	○	○	○

391. Please provide any additional items or comments related to this section.

If the person is in a severe psychotic state and needs to go to hospital

* 392. If the person needs to go to hospital, the first aider should encourage the person to go voluntarily.

- ○ Essential
- ○ Important
- ○ Don't know/depends
- ○ Unimportant
- ○ Should not be included

* 393. If admission to hospital is recommended by a mental health professional and the person does not agree, the first aider should see if one of the person’s relatives or friends can persuade them.

- ○ Essential
- ○ Important
- ○ Don't know/depends
- ○ Unimportant
- ○ Should not be included

* 394. If the person is willing to go to hospital, the first aider should offer to accompany them.

- Essential
- Important
- Don't know/depends
- Unimportant
- Should not be included

* 395. If the person goes to hospital, and it is appropriate to the relationship, the first aider should try to speak directly to the doctor or emergency staff to provide information relevant to the person's situation.

- Essential
- Important
- Don't know/depends
- Unimportant
- Should not be included

* 396. If the first aider is a family member, they should be prepared to seek involuntary treatment for the person if necessary.

- Essential
- Important
- Don't know/depends
- Unimportant
- Should not be included

* 397. The first aider should be aware of local laws relating to involuntary treatment.

- Essential
- Important
- Don't know/depends
- Unimportant
- Should not be included

* 398. If the person needs to be admitted to hospital, the first aider should support them by focusing conversation on how a hospital stay may bring relief through reducing their symptoms.

- Essential
- Important
- Don't know/depends
- Unimportant
- Should not be included

399. Please provide any additional items or comments related to this section.

AGGRESSION (crisis situation)

This section contains statements that pertain only to situations in which the person is behaving aggressively

Please rate how important (from 'essential' to 'should not be included') you think it is that each statement be included in the guidelines.

Please keep our definitions in mind when responding to this section. You can access the definitions here.

There are 5 parts to this section:

- What the first aider should know about psychosis and aggression
- How to respond if the person is being aggressive
- De-escalation when the person is behaving aggressively
- Safety considerations when the person is behaving aggressively
- Calling for help if the person is behaving aggressively

What the first aider should know about psychosis and aggression

* 400. The first aider should be aware that people with psychosis are not usually aggressive and are at a much higher risk of harming themselves than others.

- Essential
- Important
- Don't know/depends
- Unimportant
- Should not be included

* 401. The first aider should know that certain symptoms of psychosis (e.g. visual or auditory hallucinations) can cause people to become aggressive.

- Essential
- Important
- Don't know/depends
- Unimportant
- Should not be included

* 402. If the first aider is not sure whether the person may become aggressive, they should assume that the person may be dangerous.

- Essential
- Important
- Don't know/depends
- Unimportant
- Should not be included

* 403. If the person becomes aggressive, the first aider should be aware that the person's aggression may be exacerbated by the first aider taking certain steps, e.g. involving the police.

- Essential
- Important
- Don't know/depends
- Unimportant
- Should not be included

404. Please provide any additional items or comments related to this section.

How to respond if the person is being aggressive

* 405. If the person threatens the first aider, the first aider should protect themselves by leaving.

- Essential
- Important
- Don't know/depends
- Unimportant
- Should not be included

* 406. If the person is behaving aggressively, the first aider should **not:**

	Essential	Important	Don't know/depends	Unimportant	Should not be included
...respond in a hostile, disciplinary, argumentative or challenging manner.	◯	◯	◯	◯	◯
...threaten the person as this may increase fear or prompt aggressive behaviour.	◯	◯	◯	◯	◯
...give in to any threats the person may make.	◯	◯	◯	◯	◯

* 407. Once the person's aggression subsides, the first aider should explain to them that they will not tolerate aggressive behaviours or threats.

◯ Essential

◯ Important

◯ Don't know/depends

◯ Unimportant

◯ Should not be included

408. Please provide any additional items or comments related to this section.

AGGRESSION (crisis situation) Continued

Please rate how important (from ‘essential’ to ‘should not be included’) you think it is that each statement be included in the guidelines.

Please keep our definitions in mind when responding to this section. You can access the definitions here.

De-escalation when the person is behaving aggressively

* 409. If the person is behaving aggressively, the first aider should:

	Essential	Important	Don't know/depends	Unimportant	Should not be included
...clearly ask the person to stop any disruptive behaviour, e.g. "please lower your voice".	○	○	○	○	○
...avoid asking the person too many questions as this can spark defensiveness and further anger.	○	○	○	○	○
...leave the person alone until they have calmed down, if it is safe to do so.	○	○	○	○	○

* 410. If the first aider has left the person alone to give them time to calm down, they should attempt to contact the person (e.g. by phoning them) to see if the person has calmed down.

- ○ Essential
- ○ Important
- ○ Don't know/depends
- ○ Unimportant
- ○ Should not be included

411. Please provide any additional items or comments related to this section.

Safety considerations when the person is behaving aggressively

* 412. If the person’s aggression escalates out of control, the first aider should remove themselves from the situation and call emergency services.

- Essential
- Important
- Don't know/depends
- Unimportant
- Should not be included

413. Please provide any additional items or comments related to this section.

Calling for help if the person is behaving aggressively

* 414. If the police are called, the first aider should tell them that the person may be experiencing psychosis and that the first aider needs the help of the police to obtain medical treatment and to control the person’s aggressive behaviour.

- Essential
- Important
- Don't know/depends
- Unimportant
- Should not be included

* 415. The first aider should let the police know whether or not the person has a weapon.

- Essential
- Important
- Don't know/depends
- Unimportant
- Should not be included

416. Please provide any additional items or comments related to this section.

SELF-CARE FOR THE FIRST AIDER

This section contains statements about what the first aider needs to know about self-care

Please rate how important (from ‘essential’ to ‘should not be included’) you think it is that each statement be included in the guidelines.

Please keep our definitions in mind when responding to this section. You can access the definitions here.

There is 1 part to this section:

Self-care for the first aider

* 417. The first aider should look after their own mental health and wellbeing.

- Essential
- Important
- Don't know/depends
- Unimportant
- Should not be included

* 418. The first aider should know that it is common to experience negative feelings (e.g. shock, fear, sadness, anger, frustration) as a result of supporting the person.

- Essential
- Important
- Don't know/depends
- Unimportant
- Should not be included

* 419. If the first aider is finding their role stressful, they should seek support for themselves (e.g. through support groups and organisations, a mental health professional or a supportive friend), while maintaining confidentiality.

- Essential
- Important
- Don't know/depends
- Unimportant
- Should not be included

* 420. The first aider should try self-help strategies to reduce any stress they experience, e.g. relaxation methods, regular exercise, sleep, healthy diet.

- Essential
- Important
- Don't know/depends
- Unimportant
- Should not be included

* 421. The first aider should not put pressure on themselves to find solutions to all the person's problems.

- Essential
- Important
- Don't know/depends
- Unimportant
- Should not be included

422. Please provide any additional items or comments related to this section.

Thank you!

Thank you for sharing your expertise and time with us.

If anything in this survey has caused you distress and you would like to talk with someone about it you can contact the appropriate crisis help line below:

Australia: Lifeline on 13 11 14

Canada: National Suicide prevention Lifeline on 1800 273 TALK (8255)

Denmark: Suicide hotline 70 201 201

Finland: SOS Crisis Centre 010 195 202

The Netherlands: Suicide hotline 113Online

New Zealand: Lifeline Aotearoa on 0800 543 354

Republic of Ireland: Samaritans on 116 123

Sweden: Suicide hotline 020 22 00 60

UK: Samaritans on 116 123

USA: National Suicide prevention Lifeline on 1800 273 TALK (8255)
