# Supplementary material for: Offering mental health first aid to a person experiencing psychosis: a Delphi study to redevelop the guidelines published in 2008
Source: BMC Psychol. 2021 Feb 12;9:29. doi: 10.1186/s40359-021-00532-7 (PMC7881488; doi:10.1186/s40359-021-00532-7)
Supplement: Supplementary file 2 — Additional file 2. Round 2 Survey. [file 40359_2021_532_MOESM2_ESM.pdf]

Introduction and Instructions

Purpose of this project
Thank you for your participation in this project so far.

The aim of this research project is to update the mental health first aid guidelines for how a member of the public should give assistance to a person who may be experiencing psychosis. These guidelines are being developed for high income Western countries.

As advised, participation in this project involves completing three rounds of online surveys. Thank you for completing the first survey in 2018. It is now time to complete the second (shorter) survey.

Instructions
Your task is to complete the questionnaire by rating each statement according to how important you believe it is for inclusion in the guidelines for providing mental health first aid to a person who may be experiencing psychosis. This involves re-rating some items from Round 1 and rating some new items.

Please keep in mind that the guidelines will be used by the general public. **The statements need to be rated according to their importance for someone WITHOUT a counselling or clinical background.**

The majority of statements in the questionnaire pertain to both adults and adolescents. There are also a number of statements that clearly pertain only to adolescents. The adolescent-specific statements have been included as they are additional considerations that only apply to adolescents.

This questionnaire should take approximately 30-60 minutes to complete. You can complete the survey in two or more sittings. Your answers are saved when you click 'Next' at the bottom of a page. This marks your page and you can begin again at a later date on the next page. Please be aware that once you have logged on and started responding you must complete the questionnaire on the same computer.

How this questionnaire was developed
The statements in this questionnaire were derived from the results of the Round 1 survey. You will note that each statement is marked as either a NEW or RERATE item:

NEW ITEMS: these are new items that were derived from the comments provided in the first survey

RE-RATE ITEMS: these are items from the first survey that were neither endorsed or rejected by the expert groups. An item is rerated when:

- 70%–79% of panel members from both expert groups rated it as essential or important, OR
- 70%-79% of one expert group and 80-100% of the other expert group rated it as essential or important

It is important to remember that we do not necessarily agree with these statements, and some may seem contradictory or controversial. The items have been included because they reflect a wide range of people's beliefs about intervention and care. Your role is to provide us with you opinion to inform the development a set of guidelines that reflect current expert opinion.

Consent to participate
It is important for you to know that participation in this study is completely voluntary. You are not under any obligation to participate and you can withdraw at any time.

We would like to thank you for your time and effort, and encourage you to provide us with feedback on this process.

Introduction and Instructions

Definitions used in this survey

Mental health first aid is the help offered to a person developing a mental health problem, experiencing a worsening of an existing mental health problem, or in a mental health crisis. The first aid is given until appropriate professional help is received or until the crisis resolves.

The person: the person who the mental health first aider is concerned may be experiencing psychosis.

The first aider: a concerned family member, friend, work colleague or member of the community, who provides help to a person who may be experiencing psychosis.

GP/Family doctor: a medical doctor based in the community who treats patients with minor or chronic illnesses and refers those with serious conditions to a specialist or hospital.

Professional/health professional: a broad range of health professionals through which a person may seek help for psychosis. This could include a mental health professional, GP/family doctor, or another health professional, e.g. allied health professional, hospital emergency staff.

Mental health professional: a health professional who is qualified to treat people who are experiencing psychosis, e.g. a psychologist, mental health nurse or psychiatrist.

Emergency services: services that respond to and deal with emergencies when they occur, e.g. emergency medical services (ambulance) or law enforcement (the police).

Mental health crisis service: services that respond to and provide immediate help during a mental health crisis and are responsible for assessing the care required by the person. Psychiatric nurses, social workers, psychiatrists and psychologists may work for a mental health crisis service.

Crisis: a person may be in a ***crisis*** associated with psychosis if:

- They are in a severe psychotic state, e.g. the person has overwhelming delusions and hallucinations, very disorganised thinking, or bizarre and disruptive behaviours. The person may appear very distressed, their behaviours may be disturbing to others, or they may behave in a way that endangers themselves or others.
- They appear to be showing aggressive behaviour. Aggressive behaviour can cause physical or emotional harm to others and may range from verbal abuse to physical abuse.
- They are experiencing suicidal thoughts or behaviours.

Overview of the questionnaire

Section 1: Recognising and acknowledging that someone may be experiencing psychosis
Section 2: Approaching the person
Section 3: Communication (non-crisis situation)
Section 4: Talking with the person (non-crisis situation)
Section 5: Communication difficulties
Section 6: Being supportive
Section 7: Substance use
Section 8: Encouraging professional help (non-crisis situation)
Section 9: If the person doesn’t want professional help (non-crisis situation)
Section 10: Hallucinations and delusions (non-crisis situation
Section 11: When the person is in crisis (severe psychotic state or behaving aggressively)
Section 12: Severe psychotic states (crisis situation)
Section 13: Aggression (crisis situation)

Information about you

* 1. What is your name? (This allows us to determine who has completed the Round 1 survey and is therefore eligible to participate in Round 2. Your name will be deleted from your data when the project is complete).

RECOGNISING AND ACKNOWLEDGING THAT SOMEONE MAY BE DEVELOPING PSYCHOSIS

This section contains statements about what the first aider needs to know about recognising and acknowledging that someone may be experiencing psychosis.

Please rate how important (from ‘essential’ to ‘should not be included’) you think it is that each statement be included in the guidelines.

Please keep our definitions in mind when responding to this section. You can access the definitions here.

There are 2 parts to this section:

- Recognising and acknowledging that someone may be experiencing psychosis
- Knowing about psychosis

Recognising and acknowledging that someone may be experiencing psychosis

* 2. The first aider should not dismiss the person's lack of motivation or interest in life as laziness, as this may be a symptom of psychosis. (RE-RATE)

- ◯ Essential
- ◯ Important
- ◯ Don't know/depends
- ◯ Unimportant
- ◯ Should not be included

* 3. The first aider should take into consideration the spiritual and cultural context of the person’s behaviours. (RE-RATE)

- ◯ Essential
- ◯ Important
- ◯ Don't know/depends
- ◯ Unimportant
- ◯ Should not be included

Knowing about psychosis

* 4. The first aider should try to learn more about psychosis, by seeking information from reputable online resources or mental health organisations. (RE-RATE)

- Essential
- Important
- Don't know/depends
- Unimportant
- Should not be included

* 5. The first aider should be aware that psychosis is not contagious. (RE-RATE)

- Essential
- Important
- Don't know/depends
- Unimportant
- Should not be included

* 6. The first aider should be aware that psychosis is not an intellectual disability. (RE-RATE)

- Essential
- Important
- Don't know/depends
- Unimportant
- Should not be included

APPROACHING THE PERSON

This section contains statements about what the first aider needs to know about when and how to approach the person about the concerns they have for them.

Please rate how important (from ‘essential’ to ‘should not be included’) you think it is that each statement be included in the guidelines.

Please keep our definitions in mind when responding to this section. You can access the definitions here.

There is 1 part to this section.

When and how to approach the person

* 7. The first aider should approach the person rather than waiting to see if they reach out for help. (RE-RATE)

- ◯ Essential
- ◯ Important
- ◯ Don't know/depends
- ◯ Unimportant
- ◯ Should not be included

* 8. The first aider should approach the person face-to-face, if possible. (RE-RATE)

- ◯ Essential
- ◯ Important
- ◯ Don't know/depends
- ◯ Unimportant
- ◯ Should not be included

* 9. The first aider should ask the person where they would like to meet. (RE-RATE)

- ◯ Essential
- ◯ Important
- ◯ Don't know/depends
- ◯ Unimportant
- ◯ Should not be included

* 10. If the person wants to talk to the first aider but the first aider does not have time to, the first aider should tell the person this and offer to meet them another time when they can give them their full attention. (RE-RATE)

- Essential
- Important
- Don't know/depends
- Unimportant
- Should not be included

* 11. The first aider should not approach the person when they are feeling frustrated by the person's behaviour. (RE-RATE)

- Essential
- Important
- Don't know/depends
- Unimportant
- Should not be included

* 12. If the first aider doesn't know the person well, they should try to engage a friend or family member of the person to help. (NEW)

- Essential
- Important
- Don't know/depends
- Unimportant
- Should not be included

* 13. If the first aider is concerned about how the person will react to their approach, they should consider having a support person nearby. (NEW)

- Essential
- Important
- Don't know/depends
- Unimportant
- Should not be included

COMMUNICATION (in a non-crisis situation)

This section contains statements about what the first aider needs to know and do when communicating with a person who may be experiencing psychosis. This section is NOT about when the person is in crisis; later sections cover communicating during a crisis.

Please rate how important (from ‘essential’ to ‘should not be included’) you think it is that each statement be included in the guidelines.

Please keep our definitions in mind when responding to this section. You can access the definitions here.

There are 3 parts to this section:

- Guidelines for good communication
- Body language
- Listening non-judgmentally

Guidelines for good communication

* 14. The first aider should ask the person simple and direct questions. (RE-RATE)

- Essential
- Important
- Don't know/depends
- Unimportant
- Should not be included

* 15. The first aider should avoid using psychiatric terms when talking to the person. (RE-RATE)

- Essential
- Important
- Don't know/depends
- Unimportant
- Should not be included

* 16. Unless the person is experiencing communication difficulties, the first aider should use everyday language rather than simplified language. (NEW)

- Essential
- Important
- Don't know/depends
- Unimportant
- Should not be included

* 17. The first aider should use the same terminology that the person uses to describe their experiences. (NEW)

- Essential
- Important
- Don't know/depends
- Unimportant
- Should not be included

Body language

* 18. The first aider should try to appear confident when talking to the person. (NEW)

- Essential
- Important
- Don't know/depends
- Unimportant
- Should not be included

* 19. The first aider should position themselves so that the person can always see their face when they are talking to them. (NEW)

- Essential
- Important
- Don't know/depends
- Unimportant
- Should not be included

Listening non-judgmentally

* 20. The first aider should recap what the person has said **in different** words to check that they have understood correctly. (RE-RATE)

- Essential
- Important
- Don't know/depends
- Unimportant
- Should not be included

* 21. The first aider should recap what the person has said to check that they have understood correctly. (NEW)

- Essential
- Important
- Don't know/depends
- Unimportant
- Should not be included

* 22. The first aider should ask the person clarifying questions to show that they are listening. (RE-RATE)

- Essential
- Important
- Don't know/depends
- Unimportant
- Should not be included

TALKING WITH THE PERSON (in a non-crisis situation)

This section contains statements about what the first aider needs to know about talking with the person about their symptoms. This section is NOT about when the person is in crisis; later sections cover talking with a person during a crisis.

Please rate how important (from ‘essential’ to ‘should not be included’) you think it is that each statement be included in the guidelines.

Please keep our definitions in mind when responding to this section. You can access the definitions here.

There are 1 part to this section:

- Talking to the person about their symptoms

Talking to the person about their symptoms

* 23. The first aider should ask the person to explain what they are experiencing in order to gain an understanding of the person's experiences from their own perspective. (RE-RATE)

- ○ Essential
- ○ Important
- ○ Don't know/depends
- ○ Unimportant
- ○ Should not be included

* 24. The first aider should ask the person whether they have noticed changes in their own behaviour. (RE-RATE)

- ○ Essential
- ○ Important
- ○ Don't know/depends
- ○ Unimportant
- ○ Should not be included

* 25. The first aider should state, using specific behavioural examples, why they are concerned about the person, e.g. "the other day you looked frightened and were saying things that I didn't understand." (RE-RATE)

- Essential
- Important
- Don't know/depends
- Unimportant
- Should not be included

* 26. The first aider should not ask too many questions as the person may find this confronting or confusing. (NEW)

- Essential
- Important
- Don't know/depends
- Unimportant
- Should not be included

* 27. The first aider should ask the person if they want to talk to family or friends about what they are experiencing. (NEW)

- Essential
- Important
- Don't know/depends
- Unimportant
- Should not be included

COMMUNICATION DIFFICULTIES

This section contains statements about what the first aider needs to know about communication difficulties that may occur and how to talk with a person who is experiencing communication difficulties.

Please rate how important (from ‘essential’ to ‘should not be included’) you think it is that each statement be included in the guidelines.

Please keep our definitions in mind when responding to this section. You can access the definitions here.

There are 2 parts to this section:

- Communication difficulties
- Helping the person to communicate

Communication difficulties

* 28. If the person is having trouble communicating, the first aider should know that their presence alone can be reassuring for the person. (RE-RATE)

- Essential
- Important
- Don't know/depends
- Unimportant
- Should not be included

* 29. The first aider should avoid strong displays of emotion, both positive and negative. (RE-RATE)

- Essential
- Important
- Don't know/depends
- Unimportant
- Should not be included

* 30. If the first aider does not understand something that the person is trying to communicate, they should ask the person to explain. (NEW)

- Essential
- Important
- Don't know/depends
- Unimportant
- Should not be included

* 31. If the first aider is having difficulty understanding what the person is saying, they should ask the person to repeat what they have said. (NEW)

- Essential
- Important
- Don't know/depends
- Unimportant
- Should not be included

Helping the person to communicate

* 32. If appropriate and feasible, the first aider should check with others who know the person for advice on the best way to communicate with them. (RE-RATE)

- Essential
- Important
- Don't know/depends
- Unimportant
- Should not be included

* 33. If the person's speech has become disorganised, the first aider should focus on the person's feelings rather than what they are trying to say. (NEW)

- Essential
- Important
- Don't know/depends
- Unimportant
- Should not be included

* 34. If the person's speech has become disorganised, the first aider should ask if there is someone who knows them well who can join in the conversation. (NEW)

- Essential
- Important
- Don't know/depends
- Unimportant
- Should not be included

* 35. If the first aider is finding conversation with the person difficult, the first aider should try to find an activity they can do together as this may help communication, e.g. go for a walk. (NEW)

- Essential
- Important
- Don't know/depends
- Unimportant
- Should not be included

BEING SUPPORTIVE

This section contains statements about what the first aider needs to know about being supportive to a person who may be experiencing psychosis.

Please rate how important (from 'essential' to 'should not be included') you think it is that each statement be included in the guidelines.

Please keep our definitions in mind when responding to this section. You can access the definitions here.

There are 3 parts to this section:

- Being supportive and understanding
- Treating the person with dignity and respect
- Encouraging other supports

Being supportive and understanding

* 36. If appropriate to the relationship, the first aider should maintain their usual interactions with the person, e.g. by involving them in social events. (RE-RATE)

◯ Essential

◯ Important

◯ Don't know/depends

◯ Unimportant

◯ Should not be included

Treating the person with dignity and respect

* 37. The first aider should be tolerant of changes in the person's behaviour, unless their behaviour becomes dangerous or inappropriate. (RE-RATE)

◯ Essential

◯ Important

◯ Don't know/depends

◯ Unimportant

◯ Should not be included

* 38. The first aider should not attempt to take over or make decisions for the person without their involvement. (RE-RATE)

- Essential
- Important
- Don't know/depends
- Unimportant
- Should not be included

Encouraging other supports

* 39. The first aider should try to determine whether the person has a supportive social network and if they do, the first aider should encourage them to use these supports. (RE-RATE)

- Essential
- Important
- Don't know/depends
- Unimportant
- Should not be included

* 40. The first aider should encourage the person to try self-help strategies, e.g. relaxation methods, physical activity, good sleep habits. (RE-RATE)

- Essential
- Important
- Don't know/depends
- Unimportant
- Should not be included

* 41. The first aider should encourage the person to engage in a healthy lifestyle, e.g. regular exercise, healthy diet, not using substances. (RE-RATE)

- Essential
- Important
- Don't know/depends
- Unimportant
- Should not be included

* 42. The first aider should let the person know that there are programs that provide support for education and employment goals, if these are important to the person. (RE-RATE)

- Essential
- Important
- Don't know/depends
- Unimportant
- Should not be included

SUBSTANCE USE

This section contains additional statements about what the first aider needs to know specifically in relation to substance use and psychosis.

Please rate how important (from ‘essential’ to ‘should not be included’) you think it is that each statement be included in the guidelines.

Please keep our definitions in mind when responding to this section. You can access the definitions here.

There is 1 part to this section.

Substance use

* 43. The first aider should discourage the person from **using** alcohol or other drugs, as these may worsen symptoms of psychosis. (RE-RATE)

- Essential
- Important
- Don't know/depends
- Unimportant
- Should not be included

* 44. The first aider should discourage the person from **misusing** alcohol or other drugs, as these may worsen symptoms of psychosis. (NEW)

- Essential
- Important
- Don't know/depends
- Unimportant
- Should not be included

* 45. The first aider should tell the person that alcohol and other drugs can make their symptoms worse. (RE-RATE)

- Essential
- Important
- Don't know/depends
- Unimportant
- Should not be included

* 46. The first aider should tell the person that **misuse of** alcohol and other drugs can make their symptoms worse. (NEW)

- Essential
- Important
- Don't know/depends
- Unimportant
- Should not be included

* 47. If the person has been using alcohol or other drugs, the first aider should not blame or lecture them about this. (NEW)

- Essential
- Important
- Don't know/depends
- Unimportant
- Should not be included

ENCOURAGING PROFESSIONAL HELP (in a non-crisis situation) Continued

This section contains statements about what the first aider needs to know about encouraging the person to seek professional help, when the person is NOT in crisis.

Please rate how important (from ‘essential’ to ‘should not be included’) you think it is that each statement be included in the guidelines.

Please keep our definitions in mind when responding to this section. You can access the definitions here.

There are 3 parts to this section:

- Encouraging professional help - general
- Providing the person with information and resources about professional help
- Supporting the person to seek professional help

Encouraging professional help - general

* 48. The first aider should encourage the person to seek professional help. (NEW)

◯ Essential

◯ Important

◯ Don't know/depends

◯ Unimportant

◯ Should not be included

* 49. The first aider should try to find out what type of professional help the person believes will help them. (RE-RATE)

◯ Essential

◯ Important

◯ Don't know/depends

◯ Unimportant

◯ Should not be included

* 50. The first aider should not pressure the person to seek professional help, unless they are concerned for the person's safety. (RE-RATE)

- Essential
- Important
- Don't know/depends
- Unimportant
- Should not be included

Providing the person with information and resources about professional help

* 51. The first aider should provide the person with a range of options for seeking professional help. (RE-RATE)

- Essential
- Important
- Don't know/depends
- Unimportant
- Should not be included

* 52. The first aider should provide the person with relevant resources (e.g. printed materials, websites, telephone numbers) and information about local services. (RE-RATE)

- Essential
- Important
- Don't know/depends
- Unimportant
- Should not be included

* 53. If the person asks for advice or suggestions regarding treatment, the first aider should tell the person that they should talk to a professional about these. (NEW)

- Essential
- Important
- Don't know/depends
- Unimportant
- Should not be included

* 54. If the first aider finds out the person is not taking their prescribed medication they should encourage them to talk to their doctor about it. (NEW)

- Essential
- Important
- Don't know/depends
- Unimportant
- Should not be included

Supporting the person to seek professional help

* 55. The first aider should explain to the person that seeking professional help does not necessarily mean they will be hospitalised, as early treatment can take place in the community. (RE-RATE)

- Essential
- Important
- Don't know/depends
- Unimportant
- Should not be included

* 56. The first aider should explain to the person that a health professional must maintain confidentiality except in limited circumstances, e.g. if the person is at risk of harming themselves or others, or if directed to by a court. (RE-RATE)

- Essential
- Important
- Don't know/depends
- Unimportant
- Should not be included

* 57. The first aider should reassure the person that health professionals will have their wellbeing and best interests in mind. (NEW)

- Essential
- Important
- Don't know/depends
- Unimportant
- Should not be included

* 58. If the person is an adolescent, the first aider should always try to engage the person's parents or guardians. (NEW)

- Essential
- Important
- Don't know/depends
- Unimportant
- Should not be included

* 59. If the person is an adolescent and the first aider has a duty of care for them, the first aider should:

	Essential	Important	Don't know/depends	Unimportant	Should not be included
...ensure that the adolescent gets an appointment to see a professional. (NEW)					
...offer to go with them when they seek professional help. (NEW)					

IF THE PERSON DOESN'T WANT PROFESSIONAL HELP (in a non-crisis situation)

This section contains statements about what the first aider needs to know about what to do when the person doesn't want professional help, and the person is NOT in crises.

Please rate how important (from 'essential' to 'should not be included') you think it is that each statement be included in the guidelines.

Please keep our definitions in mind when responding to this section. You can access the definitions here.

There is 1 part to this section:

- If the person doesn't want professional help

If the person doesn't want professional help

* 60. If the person does not want to seek professional help, the first aider should discuss their concerns with the person using examples of behaviour or problems they have noticed. (RE-RATE)

- Essential
- Important
- Don't know/depends
- Unimportant
- Should not be included

* 61. The first aider should ask the person what they think the pros and cons of seeking professional help would be. (NEW)

- Essential
- Important
- Don't know/depends
- Unimportant
- Should not be included

HALLUCINATIONS AND DELUSIONS (in a non-crisis situation)

This section contains statements about what the first aider needs to know about hallucinations and delusions, and how to respond to them. It specifically relates to situations in which a person is or has been experiencing hallucinations or delusion but is NOT in crisis.

Please rate how important (from 'essential' to 'should not be included') you think it is that each statement be included in the guidelines.

Please keep our definitions in mind when responding to this section. You can access the definitions here.

There are 3 parts to this section:

- Finding out about what the person is experiencing
- Responding to hallucinations and delusions
- If the person is paranoid

Finding out about what the person is experiencing

* 62. The first aider should ask the person if they want to talk about what they are seeing or hearing. (RE-RATE)

- Essential
- Important
- Don't know/depends
- Unimportant
- Should not be included

* 63. The first aider should ask questions about the content of the person's delusions, particularly any elements that indicate the potential for harming themselves or others. (RE-RATE)

- Essential
- Important
- Don't know/depends
- Unimportant
- Should not be included

Responding to hallucinations and delusions

* 64. The first aider should acknowledge to the person that what they are experiencing is real to them, without confirming or denying their hallucinations or delusions, e.g. by stating "I accept that you hear voices or see things in that way, but it's not like that for me." (RE-RATE)

- Essential
- Important
- Don't know/depends
- Unimportant
- Should not be included

* 65. The first aider should let the person know that many people experience hearing voices. (RE-RATE)

- Essential
- Important
- Don't know/depends
- Unimportant
- Should not be included

* 66. The first aider should know that it is not helpful to encourage the person to try to stop hallucinations. (RE-RATE)

- Essential
- Important
- Don't know/depends
- Unimportant
- Should not be included

* 67. If it is appropriate to their relationship, the first aider should let the person know they love and support them, as this can help the person to feel safe. (RE-RATE)

- Essential
- Important
- Don't know/depends
- Unimportant
- Should not be included

* 68. If there are aspects of the person's environment that seem to increase their hallucinations or delusions, the first aider should limit or remove these where possible. (RE-RATE)

- Essential
- Important
- Don't know/depends
- Unimportant
- Should not be included

* 69. The first aider should use the person's own terminology when referring to hallucinations or delusions, e.g. 'the voices', or 'your worries about your safety'. (NEW)

- Essential
- Important
- Don't know/depends
- Unimportant
- Should not be included

If the person is paranoid

* 70. If the person is experiencing paranoia, the first aider should:

	Essential	Important	Don't know/depends	Unimportant	Should not be included
...ask the person about their fears. (RE-RATE)					
...give the person simple directions, if needed, e.g. "sit down, and let's talk about it". (RE-RATE)					
...encourage and support the person to move away from whatever is causing their fear, if it is safe to do so. (RE-RATE)					
...tell the person that they themselves do not see any threats but that they will stay with the person if it helps them feel safe. (NEW)					

* 71. The first aider should try not to use body language that could exacerbate paranoia, e.g. approach the person with hands in pockets or behind back, standing over or too close to the person. (NEW)

- Essential
- Important
- Don't know/depends
- Unimportant
- Should not be included

WHEN THE PERSON IS IN CRISIS (is in a severe psychotic state OR behaving aggressively)

This section contains statements about what the first aider needs to know about what to do when the person is in crisis (that is, they are in a severe psychotic state or are behaving aggressively, but the first aider does not think the person is at risk of suicide).

Please rate how important (from ‘essential’ to ‘should not be included’) you think it is that each statement be included in the guidelines.

Please keep our definitions in mind when responding to this section. You can access the definitions here.

There are 5 parts to this section:

- Safety considerations when the person is in a severe psychotic state or behaving aggressively
- Communicating with the person when they are in a severe psychotic state or behaving aggressively
- De-escalation when the person is in a severe psychotic state or behaving aggressively
- Seeking help for the person when they are in a severe psychotic state or behaving aggressively
- Calling emergency services for help when the person is in a severe psychotic state or behaving aggressively

Safety considerations when the person is in a severe psychotic state or behaving aggressively

* 72. The first aider should comply with requests, unless they are unreasonable or unsafe, as this gives the person the opportunity to feel somewhat in control. (RE-RATE)

	Essential	Important	Don't know/depends	Unimportant	Should not be included
Severe psychotic state	○	○	○	○	○
Behaving aggressively	○	○	○	○	○

* 73. If the first aider is alone with the person, they should call another person to accompany the first aider until professional help arrives. (RE-RATE)

	Essential	Important	Don't know/depends	Unimportant	Should not be included
Behaving aggressively	○	○	○	○	○

* 74. If the first aider is alone with the person and cannot stay, they should call someone to stay with the person until professional help arrives. (RE-RATE)

	Essential	Important	Don't know/depends	Unimportant	Should not be included
Behaving aggressively	○	○	○	○	○

* 75. If the first aider is frightened, they should tell the person this as it may help to de-escalate the situation. (NEW)

	Essential	Important	Don't know/depends	Unimportant	Should not be included
Severe psychotic state	○	○	○	○	○
Behaving aggressively	○	○	○	○	○

* 76. The first aider should try to ensure that the person has clear access to an exit. (NEW)

	Essential	Important	Don't know/depends	Unimportant	Should not be included
Severe psychotic state	○	○	○	○	○
Behaving aggressively	○	○	○	○	○

Communicating with the person when they are in a severe psychotic state or behaving aggressively

* 77. The first aider should try to minimise the level of emotion they show. (RE-RATE)

	Essential	Important	Don't know/depends	Unimportant	Should not be included
Severe psychotic state	○	○	○	○	○
Behaving aggressively	○	○	○	○	○

* 78. The first aider should position themselves at the level of the person if it is safe to do so. (RE-RATE)

	Essential	Important	Don't know/depends	Unimportant	Should not be included
Severe psychotic state	○	○	○	○	○
Behaving aggressively	○	○	○	○	○

De-escalation when the person is in a severe psychotic state or behaving aggressively

* 79. The first aider should try to de-escalate the situation. (RE-RATE)

	Essential	Important	Don't know/depends	Unimportant	Should not be included
Severe psychotic state	○	○	○	○	○
Behaving aggressively	○	○	○	○	○

* 80. The first aider should listen to the person more than talking to them. (RE-RATE)

	Essential	Important	Don't know/depends	Unimportant	Should not be included
Behaving aggressively	◯	◯	◯	◯	◯

* 81. If there are people present who do not have a role in helping with the crisis, the first aider should ask them to leave. (RE-RATE)

	Essential	Important	Don't know/depends	Unimportant	Should not be included
Severe psychotic state	◯	◯	◯	◯	◯
Behaving aggressively	◯	◯	◯	◯	◯

* 82. If the person is consuming alcohol or other drugs, the first aider should discourage them from taking any more. (RE-RATE)

	Essential	Important	Don't know/depends	Unimportant	Should not be included
Severe psychotic state	◯	◯	◯	◯	◯
Behaving aggressively	◯	◯	◯	◯	◯

WHEN THE PERSON IS IN CRISIS (is in a severe psychotic state OR behaving aggressively) Continued

Please rate how important (from 'essential' to 'should not be included') you think it is that each statement be included in the guidelines.

Please keep our definitions in mind when responding to this section. You can access the definitions here.

Seeking help for the person when they are in a severe psychotic state or behaving aggressively

* 83. If the person is receiving professional help for psychosis, the first aider should contact the person's health professional immediately. (RE-RATE)

	Essential	Important	Don't know/depends	Unimportant	Should not be included
Severe psychotic state	○	○	○	○	○
Behaving aggressively	○	○	○	○	○

* 84. If the person is receiving professional help for psychosis, the first aider should contact the person's health professional **if they have their permission.** (NEW)

	Essential	Important	Don't know/depends	Unimportant	Should not be included
Severe psychotic state	○	○	○	○	○
Behaving aggressively	○	○	○	○	○

* 85. If the person is receiving professional help for psychosis, the first aider should **encourage the person to** contact their health professional immediately. (NEW)

	Essential	Important	Don't know/depends	Unimportant	Should not be included
Severe psychotic state	○	○	○	○	○
Behaving aggressively	○	○	○	○	○

* 86. If the person threatens to harm themselves or others, the first aider should not attempt to drive the person to the hospital **without the support of others.** (RE-RATE)

	Essential	Important	Don't know/depends	Unimportant	Should not be included
Severe psychotic state	○	○	○	○	○
Behaving aggressively	○	○	○	○	○

* 87. If the person threatens to harm themselves or others, the first aider should not attempt to drive the person to the hospital. (NEW)

	Essential	Important	Don't know/depends	Unimportant	Should not be included
Severe psychotic state	○	○	○	○	○
Behaving aggressively	○	○	○	○	○

Calling for help when the person is in a severe psychotic state or behaving aggressively

* 88. If the first aider calls emergency services they should explain that they are concerned the person may be experiencing psychosis. (RE-RATE)

	Essential	Important	Don't know/depends	Unimportant	Should not be included
Severe psychotic state	○	○	○	○	○
Behaving aggressively	○	○	○	○	○

* 89. If the first aider calls emergency services and the police respond, the first aider should be prepared that the person may be restrained or face charges. (RE-RATE)

	Essential	Important	Don't know/depends	Unimportant	Should not be included
Severe psychotic state	○	○	○	○	○
Behaving aggressively	○	○	○	○	○

SEVERE PSYCHOTIC STATES (crisis situation)

This section contains statements that pertain only to severe psychotic states

Please rate how important (from 'essential' to 'should not be included') you think it is that each statement be included in the guidelines.

Please keep our definitions in mind when responding to this section. You can access the definitions here.

There are 4 parts to this section

- Communicating with the person when they are in a severe psychotic state or behaving aggressively
- De-escalation when the person is in a severe psychotic state
- If the person doesn't want professional help but is in a severe psychotic state
- If the person is in a severe psychotic state and needs to go to hospital

Communicating with the person when they are in a severe psychotic state

* 90. The first aider should know that severe psychosis may affect the person's level of comprehension and capacity to reason. (NEW)

◯ Essential

◯ Important

◯ Don't know/depends

◯ Unimportant

◯ Should not be included

De-escalation when the person is in a severe psychotic state

* 91. If the person has unrealistic fears for their safety, the first aider should try to demonstrate to the person that they are safe, e.g. by showing them that nobody is in the room if they are experiencing delusions about this. (NEW)

◯ Essential

◯ Important

◯ Don't know/depends

◯ Unimportant

◯ Should not be included

* 92. If the person has unrealistic fears for their safety, the first aider should reassure them that they are safe. (RE-RATE)

- Essential
- Important
- Don't know/depends
- Unimportant
- Should not be included

If the person doesn’t want professional help but is in a severe psychotic state

* 93. If the person is in a severe psychotic state and denies that they are unwell, the first aider should contact emergency services. (RE-RATE)

- Essential
- Important
- Don't know/depends
- Unimportant
- Should not be included

If the person is in a severe psychotic state and needs to go to hospital

* 94. If the person needs to go to hospital, the first aider should encourage the person to go voluntarily. (RE-RATE)

- Essential
- Important
- Don't know/depends
- Unimportant
- Should not be included

* 95. The first aider should be aware of local laws relating to involuntary treatment. (RE-RATE)

- Essential
- Important
- Don't know/depends
- Unimportant
- Should not be included

This section contains statements that pertain only to situations in which the person is behaving aggressively

Please rate how important (from ‘essential’ to ‘should not be included’) you think it is that each statement be included in the guidelines.

Please keep our definitions in mind when responding to this section. You can access the definitions here.

There are 2 parts to this section

- What the first aider should know about psychosis and aggression
- De-escalation when the person is behaving aggressively

What the first aider should know about psychosis and aggression

* 96. The first aider should not make assumptions about whether the person will be aggressive or not, but be aware of potential risks to others. (NEW)

- Essential
- Important
- Don't know/depends
- Unimportant
- Should not be included

* 97. The first aider should understand that the person's aggression may be driven by fear. (NEW)

- Essential
- Important
- Don't know/depends
- Unimportant
- Should not be included

De-escalation when the person is behaving aggressively

* 98. The first aider should ask the person if they would like to be left alone to calm down. (NEW)

- Essential
- Important
- Don't know/depends
- Unimportant
- Should not be included

* 99. The first aider should maintain some physical distance from the person but not leave them alone. (NEW)

- Essential
- Important
- Don't know/depends
- Unimportant
- Should not be included

Thank you!

Thank you for sharing your expertise and time with us. We will be in touch in the coming months with the third and final survey.

If anything in this survey has caused you distress and you would like to talk with someone about it you can contact the appropriate crisis help line below:

Australia: Lifeline on 13 11 14

Canada: National Suicide prevention Lifeline on 1800 273 TALK (8255)

Denmark: Suicide hotline 70 201 201

Finland: SOS Crisis Centre 010 195 202

Germany: TelefonSeelsorg 0800/111 0 111 or 0800/111 0 222 or 116 123

France: Suicide Écoute 01 45 39 40 00

The Netherlands: Suicide hotline 0900 0113

New Zealand: Lifeline Aotearoa on 0800 543 354

Republic of Ireland: Samaritans on 116 123

Sweden: Suicide hotline 020 22 00 60

Switzerland: PARSPAS 027 321 21 21

UK: Samaritans on 116 123

USA: National Suicide prevention Lifeline on 1800 273 TALK (8255)
