# Supplementary material for: Offering mental health first aid to a person experiencing psychosis: a Delphi study to redevelop the guidelines published in 2008
Source: BMC Psychol. 2021 Feb 12;9:29. doi: 10.1186/s40359-021-00532-7 (PMC7881488; doi:10.1186/s40359-021-00532-7)
Supplement: Supplementary file 3 — Additional file 3. Round 3 Survey. [file 40359_2021_532_MOESM3_ESM.pdf]

Introduction and Instructions

Purpose of this project

Thank you for your participation in this project so far.

The aim of this research project is to update the mental health first aid guidelines for how a member of the public should give assistance to a person who may be experiencing psychosis. These guidelines are being developed for high income Western countries.

As advised, participation in this project involves completing three rounds of online surveys. Thank you for completing the first two surveys. It is now time to complete the third and final survey. There are only 6 items to be re-rated in this survey.

Instructions

Your task is to complete the questionnaire by rating each statement according to how important you believe it is for inclusion in the guidelines for providing mental health first aid to a person who may be experiencing psychosis.

Please keep in mind that the guidelines will be used by the general public. **The statements need to be rated according to their importance for someone WITHOUT a counselling or clinical background.**

The statements in the questionnaire pertain to both adults and adolescents.

This questionnaire should take approximately 5 minutes to complete. You can complete the survey in two or more sittings. Your answers are saved when you click 'Next' at the bottom of the page. This marks your page and you can begin again at a later date on the next page. Please be aware that once you have logged on and started responding you must complete the questionnaire on the same computer.

How this questionnaire was developed

The statements in this questionnaire are items that were 'new' in the Round 2 survey that were neither endorsed or rejected by the expert groups, and therefore need to be re-rated.

An item is re-rated when:

- 70%-79% of panel members from both expert groups rated it as essential or important, OR
- 70%-79% of one expert group and 80-100% of the other expert group rated it as essential or important

It is important to remember that we do not necessarily agree with these statements, and some may seem contradictory or controversial. The items have been included because they reflect a wide range of people's beliefs about intervention and care. Your role is to provide us with you opinion to inform the development a set of guidelines that reflect current expert opinion.

Consent to participate

It is important for you to know that participation in this study is completely voluntary. You are not under any obligation to participate and you can withdraw at any time.

We would like to thank you for your time and effort, and encourage you to provide us with feedback on this process.

Introduction and Instructions

Definitions used in this survey

Mental health first aid is the help offered to a person developing a mental health problem, experiencing a worsening of an existing mental health problem, or in a mental health crisis. The first aid is given until appropriate professional help is received or until the crisis resolves.

The person: the person who the mental health first aider is concerned may be experiencing psychosis.

The first aider: a concerned family member, friend, work colleague or member of the community, who provides help to a person who may be experiencing psychosis.

GP/Family doctor: a medical doctor based in the community who treats patients with minor or chronic illnesses and refers those with serious conditions to a specialist or hospital.

Professional/health professional: a broad range of health professionals through which a person may seek help for psychosis. This could include a mental health professional, GP/family doctor, or another health professional, e.g. allied health professional, hospital emergency staff.

Mental health professional: a health professional who is qualified to treat people who are experiencing psychosis, e.g. a psychologist, mental health nurse or psychiatrist.

Emergency services: services that respond to and deal with emergencies when they occur, e.g. emergency medical services (ambulance) or law enforcement (the police).

Mental health crisis service: services that respond to and provide immediate help during a mental health crisis and are responsible for assessing the care required by the person. Psychiatric nurses, social workers, psychiatrists and psychologists may work for a mental health crisis service.

Crisis: a person may be in a ***crisis*** associated with psychosis if:

- They are in a severe psychotic state, e.g. the person has overwhelming delusions and hallucinations, very disorganised thinking, or bizarre and disruptive behaviours. The person may appear very distressed, their behaviours may be disturbing to others, or they may behave in a way that endangers themselves or others.
- They appear to be showing aggressive behaviour. Aggressive behaviour can cause physical or emotional harm to others and may range from verbal abuse to physical abuse.
- They are experiencing suicidal thoughts or behaviours.

Overview of the questionnaire

Section 1: Communication (non-crisis situation)
Section 2: Talking with the person (non-crisis situation)
Section 3: Communication difficulties
Section 4: Substance use
Section 5: Encouraging professional help (non-crisis situation)

Information about you

* 1. What is your name? (This allows us to determine who has completed the survey). Your name will be deleted from your data when the project is complete).

COMMUNICATION (in a non-crisis situation)

This section contains statements about what the first aider needs to know and do when communicating with a person who may be experiencing psychosis. This section is NOT about when the person is in crisis; later sections cover communicating during a crisis.

Please rate how important (from ‘essential’ to ‘should not be included’) you think it is that each statement be included in the guidelines.

Please keep our definitions in mind when responding to this section. You can access the definitions here.

There is 1 part to this section.

Body language

* 2. The first aider should try to appear confident when talking to the person.

- Essential
- Important
- Don't know/depends
- Unimportant
- Should not be included

TALKING WITH THE PERSON (in a non-crisis situation)

This section contains statements about what the first aider needs to know about talking with the person about their symptoms. This section is NOT about when the person is in crisis; later sections cover talking with a person during a crisis.

Please rate how important (from ‘essential’ to ‘should not be included’) you think it is that each statement be included in the guidelines.

Please keep our definitions in mind when responding to this section. You can access the definitions here.

There are 1 part to this section.

Talking to the person about their symptoms

* 3. The first aider should not ask too many questions as the person may find this confronting or confusing.

- ◯ Essential
- ◯ Important
- ◯ Don't know/depends
- ◯ Unimportant
- ◯ Should not be included

COMMUNICATION DIFFICULTIES

This section contains statements about what the first aider needs to know about communication difficulties that may occur and how to talk with a person who is experiencing communication difficulties.

Please rate how important (from ‘essential’ to ‘should not be included’) you think it is that each statement be included in the guidelines.

Please keep our definitions in mind when responding to this section. You can access the definitions here.

There is 1 part to this section.

Helping the person to communicate

* 4. If the person's speech has become disorganised, the first aider should focus on the person's feelings rather than what they are trying to say.

- ◯ Essential
- ◯ Important
- ◯ Don't know/depends
- ◯ Unimportant
- ◯ Should not be included

SUBSTANCE USE

This section contains additional statements about what the first aider needs to know specifically in relation to substance use and psychosis.

Please rate how important (from ‘essential’ to ‘should not be included’) you think it is that each statement be included in the guidelines.

Please keep our definitions in mind when responding to this section. You can access the definitions here.

There is 1 part to this section.

Substance use

* 5. The first aider should discourage the person from **misusing** alcohol or other drugs, as these may worsen symptoms of psychosis.

- ◯ Essential
- ◯ Important
- ◯ Don't know/depends
- ◯ Unimportant
- ◯ Should not be included

* 6. The first aider should tell the person that **misuse of** alcohol and other drugs can make their symptoms worse.

- ◯ Essential
- ◯ Important
- ◯ Don't know/depends
- ◯ Unimportant
- ◯ Should not be included

ENCOURAGING PROFESSIONAL HELP (in a non-crisis situation) Continued

This section contains statements about what the first aider needs to know about encouraging the person to seek professional help, when the person is NOT in crisis.

Please rate how important (from ‘essential’ to ‘should not be included’) you think it is that each statement be included in the guidelines.

Please keep our definitions in mind when responding to this section. You can access the definitions here.

There is 1 part to this section.

Encouraging professional help - general

* 7. The first aider should encourage the person to seek professional help.

- ◯ Essential
- ◯ Important
- ◯ Don't know/depends
- ◯ Unimportant
- ◯ Should not be included

Thank you!

Thank you for sharing your expertise and time with us. We will be in touch in the coming months with the third and final survey.

If anything in this survey has caused you distress and you would like to talk with someone about it you can contact the appropriate crisis help line below:

Australia: Lifeline on 13 11 14

Canada: National Suicide prevention Lifeline on 1800 273 TALK (8255)

Denmark: Suicide hotline 70 201 201

Finland: SOS Crisis Centre 010 195 202

Germany: TelefonSeelsorg 0800/111 0 111 or 0800/111 0 222 or 116 123

France: Suicide Écoute 01 45 39 40 00

The Netherlands: Suicide hotline 0900 0113

New Zealand: Lifeline Aotearoa on 0800 543 354

Republic of Ireland: Samaritans on 116 123

Sweden: Suicide hotline 020 22 00 60

Switzerland: PARSPAS 027 321 21 21

UK: Samaritans on 116 123

USA: National Suicide prevention Lifeline on 1800 273 TALK (8255)
